# Clinical Outcomes and Non‐Invasive Testing in Metabolic Dysfunction‐Associated Steatohepatitis With Cirrhosis: A Systematic Review

**DOI:** 10.1111/liv.70608

**Published:** 2026-03-28

**Authors:** Hannes Hagström, Chris Hellmund, Jeffrey V. Lazarus, Riku Ota, Mary E. Rinella, Giada Sebastiani, Fotis Tefos, Zobair M. Younossi, Mazen Noureddin

**Affiliations:** ^1^ Department of Medicine, Huddinge Karolinska Institutet Stockholm Sweden; ^2^ Division of Hepatology, Department of Upper GI Diseases Karolinska University Hospital Stockholm Sweden; ^3^ Oxford PharmaGenesis Oxford UK; ^4^ CUNY Graduate School of Public Health and Health Policy New York City New York USA; ^5^ Barcelona Institute for Global Health (ISGlobal) Barcelona Spain; ^6^ Novo Nordisk A/S Søborg Denmark; ^7^ University of Chicago Pritzker School of Medicine Chicago Illinois USA; ^8^ Division of Gastroenterology and Hepatology, Department of Medicine McGill University Health Centre Montreal Quebec Canada; ^9^ The Global NASH/MASH Council Washington DC USA; ^10^ Beatty Liver and Obesity Research Program Inova Health System Falls Church Virginia USA; ^11^ Houston Research Institute Houston Texas USA; ^12^ Houston Methodist Hospital Houston Texas USA

**Keywords:** healthcare outcome assessment, liver cirrhosis, metabolic dysfunction‐associated steatohepatitis, systematic reviews

## Abstract

**Background and Aims:**

Metabolic dysfunction‐associated steatohepatitis (MASH) with cirrhosis lacks definitive treatments and poses an increasing healthcare burden globally. We undertook a systematic literature review (SLR) to better understand the disease burden in cirrhosis due to MASH.

**Methods:**

The SLR was conducted according to Preferred Reporting Items for Systematic Reviews and Meta‐Analyses guidelines and registered with PROSPERO (CRD4202458650). Embase, MEDLINE, and the Cochrane Library were searched for clinical trials, observational studies, and SLRs/meta‐analyses published in 2014–2024 in MASH‐related cirrhosis. This study focuses on clinical outcomes, including the role of non‐invasive tests (NITs) for predicting these outcomes in patients with MASH‐related cirrhosis.

**Results:**

Following full‐text review, 317 studies were considered eligible for inclusion. Studies on transplant‐free survival, decompensation events, and hepatocellular carcinoma (HCC) in cirrhosis due to MASH were few and heterogeneous. In patients with MASH and compensated cirrhosis, transplant‐free survival was lower in patients with type 2 diabetes (T2D) than in those without. Risks of decompensation increased with factors that included the presence of varices and T2D. Male sex, T2D, and high Child–Turcotte–Pugh score were risk factors for developing HCC. Three studies compared the performance of NITs for predicting liver‐related events, including decompensation, but used varying definitions for these outcomes.

**Conclusions:**

This SLR identified data on a range of clinical outcomes in patients with MASH‐related cirrhosis. However, there was limited evidence for certain outcomes including the role of prognostic prediction models for liver‐related events in this patient population.

## Key Points

- A better understanding of clinical outcomes and the disease burden of metabolic dysfunction‐associated steatohepatitis (MASH) with cirrhosis will help us to estimate the potential impact of future treatments.
- Here, we report clinical outcomes specific to MASH with cirrhosis identified via a systematic review of the literature, published from 2014 to 2024.
- Few included studies reported data on survival without transplant, decompensation, factors linked to the risk of developing hepatocellular carcinoma, and prediction of cirrhosis‐related complications using non‐invasive tests (NITs).
- We also identified evidence gaps, including the relative performance of different NITs for predicting cirrhosis‐related complications.

**Abbreviations**
aHR: adjusted hazard ratio
AIH: autoimmune hepatitis
ALBI: albumin‐bilirubin index
ALD: alcohol‐related liver disease
ALT: alanine transaminase
APRI: aspartate transaminase to platelet ratio index
AST: aspartate transaminase
AUROC: area under the receiver operator characteristic curve
C: concordance
CI: confidence interval
CTP: Child–Turcotte–Pugh
eGFR: estimated glomerular filtration rate
ELF: Enhanced Liver Fibrosis
FIB‐4: fibrosis‐4 index
HbA1c: glycated haemoglobin
HCC: hepatocellular carcinoma
HR: hazard ratio
ICD: International Classification of Diseases
INR: international normalised ratio
LSM: liver stiffness measurement
MASH: metabolic dysfunction‐associated steatohepatitis
MASLD: metabolic dysfunction‐associated steatotic liver disease
MELD: Model for End‐Stage Liver Disease
NASH: non‐alcoholic steatohepatitis
NFS: non‐alcoholic fatty liver disease fibrosis score
NIT: non‐invasive test
NPV: negative predictive value
OR: odds ratio
PPV: positive predictive value
PRISMA: Preferred Reporting Items for Systematic Reviews and Meta‐Analyses
PY: person‐year
RCT: randomised controlled trial
SHR: subdistribution hazard ratio
sHR: sub‐hazard ratio
SLR: systematic literature review
T2D: type 2 diabetes
VCTE: vibration‐controlled transient elastography

## 1 Introduction

Metabolic dysfunction‐associated steatotic liver disease (MASLD) is the most common chronic liver disease, affecting an estimated 38% of adults globally in the period 2016 to 2019 [1]. Between 12 and 40% of patients with MASLD progress to metabolic dysfunction‐associated steatohepatitis (MASH), which is characterised by hepatocellular ballooning and lobular inflammation that, over time, may lead to fibrosis, the most severe stage of which is F4 or cirrhosis [2, 3]. The lifetime costs of liver complications due to MASH are substantial, estimated at $222.6 billion for all patients with MASH in the USA in 2017 [4]. In Europe, the total economic cost of MASH in 2018 was reported to be €8.5–19.5 billion [5].

Approximately one‐fifth of adults with MASH and fibrosis stage F3 progress to cirrhosis (F4) over a 2‐year period [6]. Complications of MASH with cirrhosis include decompensation events and hepatocellular carcinoma (HCC); these complications can negatively impact quality of life, lead to considerable mortality, and result in further economic burden on healthcare systems [7]. In the USA, healthcare resource utilisation and healthcare costs in patients with MASH have been estimated to be 3.9 times greater for those with cirrhosis than without [7].

Liver transplant is also a substantial contributor to healthcare resource use in patients with MASH; the proportion of liver transplants performed annually in patients with MASH in the USA increased from 2.5% in 2004 to 20.4% in 2019; in Europe from 1.2% in 2002 to 8.4% in 2016; and in Japan from 2% in 2007 to 11.5% in 2017 [8]. In candidates for liver transplant in the USA, MASH was reported as the leading cause of HCC in 2024 [9]. Cirrhosis due to MASH was reported as the leading cause of liver transplant in women in the USA in 2018 [10], and in 2023, MASH was projected to become the most common indication for liver transplant within a decade globally [8].

Given the increasing healthcare burden of MASH with cirrhosis worldwide, there is a pressing need for definitive treatments for the condition. The US Food and Drug Administration has approved resmetirom and semaglutide for MASH, but these treatments are indicated in combination with diet and exercise in adults with moderate to advanced liver fibrosis (stages F2 to F3) only [11, 12]. Several candidate pharmacotherapies are under investigation in clinical trials for MASH with cirrhosis (stage F4) [13], and so a clearer understanding of clinical outcomes and the disease burden in this specific patient population will help us to understand the potential impact of these treatments.

We conducted an extensive systematic literature review (SLR) to identify studies reporting data on clinical outcomes, humanistic and economic burden in patients with cirrhosis due to MASH. In this review, we focus on clinical outcomes of survival, decompensation and development of HCC in patients with cirrhosis due to MASH.

## 2 Materials and Methods

### 2.1 Literature Search

This SLR was designed to identify relevant data on the clinical, humanistic and economic burden of MASH with cirrhosis; the findings linked to humanistic and economic burden will be presented elsewhere. The key clinical outcomes we sought to focus on were survival and liver‐related events, including decompensation and HCC in patients with cirrhosis due to MASH. The study protocol was developed, and the SLR was conducted in line with the 2020 Preferred Reporting Items for Systematic Reviews and Meta‐Analyses (PRISMA) guidelines (see Data S1) [14] and registered with PROSPERO (registration number: CRD42024586520).

Electronic searches were conducted via Ovid on 25 July 2024 in Embase (from 1974), MEDLINE and MEDLINE In‐Process (from 1946), and the Cochrane Library using a mixture of free text and Medical Subject Headings (MeSH) terms (Tables S1–S3) to identify relevant peer‐reviewed literature published since 1 January 2014. Non‐alcoholic steatohepatitis (NASH) was renamed ‘MASH’ following a consensus statement from international experts in 2023 to reflect the updated understanding of its disease mechanism and to reduce the stigma associated with the previous terminology [15]. Accordingly, the term ‘NASH’ was also included in the searches so that all relevant studies could be captured (Tables S1–S3).

Search results were exported into a table in Microsoft Excel via EndNoteX9 and deduplicated manually; no other software and no artificial intelligence tools were employed in the development of this SLR.

### 2.2 Screening and Full‐Text Review

The titles and abstracts of studies identified by the searches were screened to determine whether the studies met the predefined eligibility criteria, including population, intervention, comparison, outcomes and study design (Table 1). For inclusion, studies were required to report at least one relevant outcome in patients with cirrhosis due to MASH (Table 1). Primary studies, SLRs, and meta‐analyses were included, but congress abstracts, case studies, case reports, and other review articles were excluded. Only English language studies were included, and there was no restriction by study geography. All studies that passed through title and abstract screening were obtained as full‐text publications and reassessed against the eligibility criteria (Table 1).

**TABLE 1:** Eligibility criteria for the systematic literature review.

Populations	Patients with MASH at stage F4 (cirrhosis including compensated and decompensated cirrhosis)
Intervention	No restriction by intervention
Comparators	Any or none
Outcomes	Clinical outcomes: Fibrosis improvement ≥ 1 stage without worsening of MASH Fibrosis improvement with no worsening of ballooning and inflammation Regression of cirrhosis in F4 Liver injury, assessed using ALT and AST Progression to liver transplantation, including by worsening in MELD score to ≥ 15 Decompensation events Hepatocellular carcinoma Variceal haemorrhage Hepatic encephalopathy Complications of ascites Mortality Use of NITs, including ELF, FAST, VCTE, FIB‐4, MELD and NAS ○ Tests used for diagnosis, monitoring and follow‐up ○ Cut‐off values used for diagnosis and severity ○ Results of tests Epidemiologic outcomes:^a^ Patient characteristics by method of MASH identification Prevalence and characteristics of sub‐populations within stage F4 Disease progression, including long‐term outcomes (including estimations) Risk factors, surrogate endpoints and comorbidities associated with the development of cirrhosis Disease reversibility or resolution Time to liver‐related outcomes in subgroups Humanistic burden, as measured by: NASH‐CHECK SF‐36 SF‐6D EQ‐5D‐5L/3L CLDQ CLDQ‐NASH WPAI:SHP Outcomes related to ADL, including fatigue (including PROMIS‐Fatigue scores), pain, functional impairment or activity limitations Economic burden, focusing on: Direct medical/non‐medical costs Indirect/societal costs Caregiver burden Healthcare resource utilisation Cost drivers, including hospitalisation and length of stay Methods of valuation
Study design	Clinical trials and observational studies Animal studies and in vitro studies were excluded
Date restrictions	2014–present^b^
Language restrictions	English language only
Publication type	All primary publications, systematic literature reviews and meta‐analyses Case studies, case reports and review articles were excluded
Country	No restriction

Abbreviations: ADL, activities of daily living; ALT, alanine transaminase; AST, aspartate transaminase; CLDQ, Chronic Liver Disease Questionnaire; CLDQ‐NASH, Chronic Liver Disease Questionnaire‐Non‐Alcoholic Steatohepatitis; ELF, Enhanced Liver Fibrosis; EQ‐5D‐5L/3L, 5‐dimension EuroQol questionnaire – five level/three level; F4, fibrosis stage 4; FAST, FibroScan‐AST; FIB‐4, fibrosis‐4 index; MASH, metabolic dysfunction‐associated steatohepatitis; MELD, Model for End‐Stage Liver Disease; NAS, non‐alcoholic fatty liver disease activity score; NASH, non‐alcoholic steatohepatitis; NIT, non‐invasive test; PROMIS, Patient Reported Outcome Measurement Information System; SF‐36, 36‐item Short‐Form Health Survey; SF‐6D, Short‐Form Six‐Dimension; VCTE, vibration‐controlled transient elastography; WPAI:SHP, Work Productivity and Activity Impairment: Specific Health Problem.

^a^ Electronic searches do not include specific terms for all epidemiologic outcomes, but any relevant studies or data on these outcomes were included.

^b^ Searches were carried out on 25 July 2024.

Owing to the large number of studies identified in preliminary electronic searches, title and abstract screening and full‐text review were split between two reviewers. Each reviewer followed the same set of instructions regarding the approach to screening. Uncertainties as to study eligibility were referred to one independent reviewer.

### 2.3 Data Extraction and Prioritisation

Owing to a high inclusion rate in the full‐text review stage, studies were prioritised systematically for data extraction. Data were extracted from studies with more than 20 patients, or more than 50 patients for studies reporting clinical outcomes, as findings of studies with very small sample sizes may have limited generalisability [16].

Publications that reported the following outcomes in isolation (i.e., those that reported no other relevant outcomes) were also considered to have limited utility and were not data extracted: (1) non‐invasive tests (NITs) that were not named specifically in the eligibility criteria shown in Table 1; (2) economic outcomes other than healthcare costs, healthcare resource utilisation and liver transplant rates; (3) epidemiology outcomes; (4) the fatigue domain of the Chronic Liver Disease Questionnaire‐NASH.

The electronic searches in this SLR did not include specific terms for all epidemiologic outcomes, but studies with relevant outcomes were included at screening and full‐text review. Epidemiologic outcomes were therefore considered exploratory, and epidemiology data from these studies were not extracted.

While SLRs were included, they were not data extracted to avoid potential duplication of extracted data from primary studies.

Detailed data, including study setting and methods, patient characteristics and relevant results, were extracted by three reviewers into a data extraction table and independently quality checked. Discrepancies were resolved by a senior reviewer who was independent from the data extraction and quality checking process.

## 3 Results

### 3.1 Search Results

In total, 4011 publications (full text and abstracts) were identified in the initial searches, of which 614 publications were removed as duplicates. Of the 3397 publications included for screening by abstract and title, 396 met the inclusion criteria for full‐text review.

**FIGURE 1:**
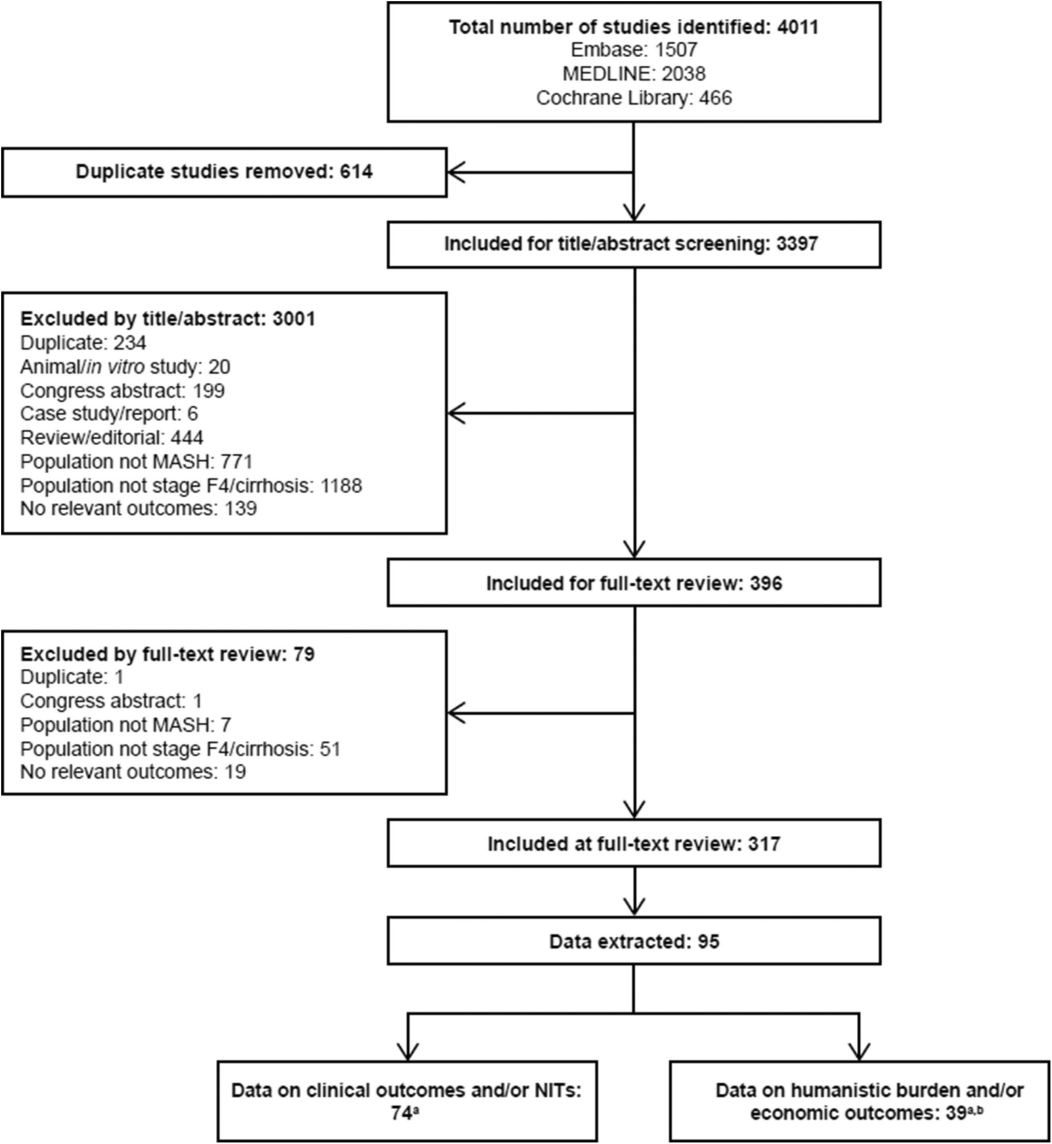
PRISMA diagram for the SLR. ^a^Some studies included both groups of outcomes. ^b^These data will be presented elsewhere. F4, fibrosis stage 4; MASH, metabolic dysfunction‐associated steatohepatitis; PRISMA, Preferred Reporting Items for Systematic Reviews and Meta‐Analyses; SLR, systematic literature review.

A total of 317 publications were eligible after full‐text review (Table S4). Data were extracted from 95 prioritised publications (Table S4), of which 74 (Table S5) had data on clinical outcomes and/or NITs, and 39 had data on humanistic burden and/or economic outcomes (see PRISMA flow diagram; Figure 1). This review focuses on the data extracted for clinical outcomes and NITs; the humanistic and economic burden data will be presented in a separate publication.

### 3.2 Data Sources

#### 3.2.1 Geography

Of the 74 studies that had data extracted for clinical outcomes and/or NITs (Table S5) [17, 18, 19, 20, 21, 22, 23, 24, 25, 26, 27, 28, 29, 30, 31, 32, 33, 34, 35, 36, 37, 38, 39, 40, 41, 42, 43, 44, 45, 46, 47, 48, 49, 50, 51, 52, 53, 54, 55, 56, 57, 58, 59, 60, 61, 62, 63, 64, 65, 66, 67, 68, 69, 70, 71, 72, 73, 74, 75, 76, 77, 78, 79, 80, 81, 82, 83, 84, 85, 86, 87, 88, 89, 90], 32 reported data from the USA [17, 19, 20, 23, 24, 25, 26, 27, 28, 32, 35, 39, 43, 44, 49, 50, 51, 52, 53, 54, 55, 57, 58, 59, 68, 69, 70, 76, 80, 81, 82, 88] and two originated from Canada [40, 56]. Three studies reported combined data from the USA and either Europe [33, 46] or the UK [22]. Two studies reported data from France and Switzerland [73, 74] and one study reported data from France and China [77]. Eight studies reported evidence from single countries across Europe: two from the UK [29, 64] and one each from Austria [41], Denmark [61], Italy [31], Germany [34], Romania [67] and Sweden [36]. Two studies reported data from Oceania (Australia) [21, 75], one from South America (Brazil [18]), and one from Africa (Egypt [62]). Ten studies reported evidence from Asia, with three studies originating from Japan [47, 63, 83], two each from China [45, 66] and Iran [30, 42], and one each from India [71], Pakistan [89] and Singapore [65]. Data from multiple countries worldwide were reported in eight studies [37, 38, 60, 72, 78, 79, 85, 90] and the country of origin was not reported in four studies [48, 84, 86, 87].

#### 3.2.2 Study Design

In total, 20 studies were prospective [22, 29, 32, 33, 37, 38, 45, 46, 52, 60, 61, 62, 64, 71, 76, 77, 79, 82, 83, 87], 46 were retrospective [18, 19, 20, 24, 25, 26, 28, 30, 31, 34, 35, 36, 39, 40, 41, 42, 43, 44, 47, 48, 49, 50, 51, 53, 54, 55, 56, 57, 58, 59, 65, 67, 68, 69, 70, 72, 73, 74, 75, 78, 80, 81, 84, 85, 86, 90] and the perspective was unclear in eight studies (Table S5) [17, 21, 23, 27, 63, 66, 88, 89]. Fifty‐seven studies were observational [17, 18, 19, 20, 21, 22, 23, 25, 26, 27, 28, 29, 30, 31, 34, 35, 36, 39, 40, 41, 42, 43, 44, 45, 47, 49, 50, 51, 52, 53, 54, 55, 56, 57, 58, 59, 61, 63, 64, 65, 66, 67, 68, 69, 70, 71, 72, 73, 74, 75, 76, 77, 80, 81, 83, 88, 89], nine were randomised controlled trials (RCTs) [32, 33, 37, 38, 46, 60, 79, 82, 87] and one was a single‐arm interventional trial [62]. Seven publications reported meta‐analyses [48, 84, 86], data from pooled analysis [78, 85, 90] or data derived from models [24].

#### 3.2.3 Study Period

A total of 57 observational studies reported study periods ranging from 1 to 31 years with a median of 10 years in duration (Table S5) [17, 18, 19, 20, 21, 22, 23, 25, 26, 27, 28, 29, 30, 31, 34, 35, 36, 39, 40, 41, 42, 43, 44, 45, 47, 49, 50, 51, 52, 53, 54, 55, 56, 57, 58, 59, 61, 63, 64, 65, 66, 67, 68, 69, 70, 71, 72, 73, 74, 75, 76, 77, 80, 81, 83, 88, 89]. Compared with observational studies, the study periods of RCTs were shorter and most RCTs had a duration of 2–3 years.

### 3.3 Overview of Key Themes

Of the 74 studies that reported on clinical outcomes and/or the use of NITs in patients with cirrhosis due to MASH, 46 studies reported clinical outcomes data [17, 19, 20, 24, 26, 27, 28, 30, 31, 32, 33, 37, 38, 40, 41, 42, 44, 46, 48, 49, 51, 52, 53, 54, 55, 56, 57, 58, 59, 60, 61, 62, 65, 68, 69, 71, 72, 73, 74, 75, 79, 80, 81, 82, 85, 87]. Key themes were survival and liver‐related events (including liver transplant), particularly decompensation and the development of HCC in patients with cirrhosis due to MASH prior to liver transplant. Another key theme was post‐transplant outcomes, including recipient survival and the recurrence of cirrhosis.

Table 2 summarises studies that report data on clinical outcomes without liver transplant in patients with cirrhosis due to MASH. Table 3 summarises the use of NITs for predicting liver‐related events, particularly decompensation, in this patient population.

**TABLE 2:** Clinical outcomes without liver transplant in patients with cirrhosis due to MASH.

Geography (study period)	Clinical context or data source (sample size)	Definition of cirrhosis	Outcome definition and incidence	Associated risks/predictors in multivariable models	Reference
*Survival, death or deterioration*					
Singapore (2004–2015)	Patients with MASH admitted to hospital for the management of first onset ascites (*n* = 99)	Based on a combination of biochemical, imaging and endoscopic features	Transplant‐free survival at 60 months, % (95% CI): 27.2 (16.1–39.6)	Factors associated with cirrhosis‐related death or liver transplant, SHR (95% CI):^a^ eGFR < 60 mL/min/1.73 m^2^ (ref: NR): 2.04 (1.13–3.68) MELD score ≥ 15 (ref: NR): 4.17 (2.05–8.47)	Tan et al. [65]
USA (2002–2016)	Adults aged ≥ 18 years with MASH on the UNOS database (*n* = 7935)	NR	Cumulative (95% CI) incidence of waitlist death or deterioration at: 2 months: 0.07 (0.06–0.07) 6 months: 0.13 (0.12–0.13) 1 year: 0.18 (0.17–0.19) 2 years: 0.25 (0.24–0.26) 3 years: 0.29 (0.28–0.30) 5 years: 0.35 (0.34–0.36)	Factors associated with increased death or deterioration, aHR (95% CI):^a^, ^b^ KPS score ≤ 30% (ref: ≥ 40%): 1.78 (1.66–1.92) Presence of encephalopathy: 1.21 (1.16–1.26) Presence of T2D: 1.11 (1.06–1.17) MELD score (ref: NR): 1.10 (1.09–1.10) Hispanic ethnicity (ref: White ethnicity): 1.13 (1.07–1.19) Serum albumin (ref: NR): 0.97 (0.97–0.98)	Thuluvath et al. [69]
Australia, Cuba, Hong Kong and Spain (1995–2016)	Patients with MASH and compensated cirrhosis who presented at hepatology clinics in tertiary referral centres (*n* = 299)	Biopsy‐defined	All‐cause deaths: *n* = 33 Transplant‐free survival at 10 years, % (95% CI) With T2D:^c^ 38 (31–45) Without T2D:^c^ 81 (75–88) Annualised mortality or transplant rates, per 100 PYs (95% CI) With T2D:^c^ 4.9 (3.76–6.37) Without T2D:^c^ 3.0 (1.81–4.98)	After adjustment for other variables,^d^ the presence of T2D^c^ increased the risk of all‐cause mortality or transplant by 4.59 (95% CI: 2.23–9.43). This remained statistically significant when T2D was analysed as a time‐dependent variable (aHR: 4.23; 95% CI: 1.93–9.29). Metformin use (as a time‐dependent variable) was associated independently with a reduced risk of all‐cause mortality (HR: 0.41; 95% CI: 0.26–0.45)	Vilar‐Gomez et al. [72]
*Decompensation events*					
USA (2015–2017)	Patients with MASH and compensated cirrhosis and portal hypertension who were enrolled in a phase 2 RCT (NCT02462967) (*n* = 162)	Biopsy‐defined	LREs were defined as at least one of the following: Development or progression of gastroesophageal varices, new‐onset ascites, variceal haemorrhage and hepatic encephalopathy An increase in CTP score of > 2 points from baseline An increase in MELD score to > 15 At 52 weeks, 33 participants (20%) had the following LREs: ascites (*n* = 17), decompensation (*n* = 13), CTP score increase of > 2 or MELD score > 15 (*n* = 3)	NR	Are et al. [82]
Australia, Cuba, Hong Kong and Spain (derivation cohort: 1995–2016) USA (validation cohort: 2004–2017)	Patients with MASH and cirrhosis with no previous episodes of clinical decompensation (derivation cohort, *n* = 299; validation cohort, *n* = 244)	Biopsy‐defined	First event of hepatic decompensation, defined by the occurrence of any of the following: ascites (identified or confirmed by abdominal ultrasound), upper gastrointestinal bleeding secondary to portal hypertension (confirmed by endoscopy in the presence of gastroesophageal varices or portal hypertensive gastropathy) or hepatic encephalopathy (established by clinical parameters, neuropsychological tests or electroencephalogram) Derivation cohort Follow‐up, median (range), years: 5.1 (2.6–8.4) Decompensation, *n* (%): 81 (27) Validation cohort Follow‐up, median (range), years: 5.4 (4.5–8.8) Decompensation, *n* (%): 132 (54)	Predictors of hepatic decompensation in the derivation cohort, sHR (95% CI):^a^ Oesophageal varices (yes; ref: no): 2.08 (1.16–3.61) T2D (yes; ref: no): 2.25 (1.20–3.58) INR (ref: NR): 6.66 (2.87–10.4) Total bilirubin (ref: NR): 1.32 (1.14–1.67) AST/ALT ratio (ref: NR): 2.28 (1.50–3.45)	Calzadilla‐Bertot et al. [24]
North America and Europe (NR)	Patients aged 18–65 years with MASH and cirrhosis who participated in a phase 2 (NCT03053063) or phase 3 RCT (NCT01672879) (*n* = 734)	Biopsy‐defined	LREs were defined as clinically apparent ascites requiring treatment, grade ≥ 2 hepatic encephalopathy according to the West Haven criteria requiring treatment and portal hypertension‐related gastrointestinal bleeding, liver transplant, qualification for transplant (MELD score ≥ 15) or mortality During median follow‐up of 16.2 months (IQR: 13.9–18.7), 4% of participants (*n* = 27) had the following LREs: ascites (*n* = 15), hepatic encephalopathy (*n* = 5), portal hypertension‐related upper gastrointestinal bleeding (*n* = 3), qualification for liver transplantation (*n* = 2), liver transplantation (*n* = 1) and death (*n* = 1)	LSM by VCTE ≥ 30.7 kPa predicted LREs with HR (95 CI%) of 10.13 (4.38–23.41). In the multivariable model, sex, ethnicity, BMI and weight loss ≥ 5% were non‐statistically significant predictors (*p* > 0.05)	Loomba et al. [85]
Australia, Cuba, Hong Kong and Spain (1995–2016)	Patients with MASH and compensated cirrhosis who presented at hepatology clinics in tertiary referral centres (*n* = 299)	Biopsy‐defined	Liver decompensation was defined by the following: occurrence of ascites (identified clinically or by ultrasound), upper gastrointestinal bleeding secondary to portal hypertension (confirmed by endoscopy in the presence of gastroesophageal varices or hypertensive gastropathy), hepatic encephalopathy (established by clinical parameters, neuropsychological tests or electroencephalogram) 83 of 299 patients developed a first event of decompensation during a median (range) follow‐up duration of 5.1 (0.5–10.0) years Ascites (60 of 83 patients) and variceal bleeding (18 of 83) were the most common first events of decompensation 10‐year cumulative incidence of decompensation, % (95% CI) With T2D:^c^ 51 (44–59) Without T2D:^c^ 26 (17–33) Annualised rates of decompensation, per 100 PYs (95% CI) With T2D:^c^ 6.6 (5.2–8.5) Without T2D:^c^ 4.2 (2.7–6.6)	Risk of decompensation in patients with T2D is 2.46 times (95% CI: 1.35–4.46) compared with those without T2D. When analysed as a time‐dependent covariate, T2D remained significantly predictive of decompensation (adjusted sHR, 2.03; 95% CI: 1.01–4.11)	Vilar‐Gomez et al. [72]
*Development of HCC*					
USA (2004–2008)	Data in the national VA Corporate Data Warehouse and VA Central Cancer Registry from patients aged ≥ 18 years with MASLD^e^ and cirrhosis (*n* = 3013)	Based on ≥ 2 outpatient or ≥ 1 inpatient ICD‐9/10 codes for cirrhosis or its complications^f^	HCC was identified based on keyword searches, or by ICD‐9/10 codes Of the 3013 patients with a diagnosis of cirrhosis at 3 years, the incidence of HCC, per 1000 PYs (95% CI) in those with FIB‐4 scores > 2.67 at baseline and 3 years was 26.62 (21.29–32.87) and 3.43 (1.88–5.76) in those with FIB‐4 scores < 1.45	NR	Cholankeril et al. [26]
USA (2002–2016)	Adults with MASLD or MASH and cirrhosis (*n* = 950)	Biopsy‐defined, or clinically diagnosed based on radiological evidence of cirrhosis and signs or symptoms of portal hypertension^g^	HCC was defined by pathological diagnosis or radiological diagnosis of Liver Imaging Reporting and Data System 5 criteria HCC developed in 82 patients (8.6%) with cirrhosis due to MASH/MASLD during the 4326 PYs of follow‐up; annual incidence: 1.90 per 100 PYs (95% CI: 1.53–2.35)	Increased risks of HCC development, HR (95% CI)^a^ Male sex (ref: NR): 4.06 (2.54–6.51) Older age (ref: NR): 1.05 (1.03–1.08) Higher CTP score (ref: NR): 1.38 (1.18–1.60) Decreased risks of HCC development, HR (95% CI)^a^ Statin use (ref: NR): 0.40 (0.24–0.67)	Pinyopornpanish et al. [54]
Australia, Cuba, Hong Kong and Spain (1995–2016)	Patients with MASH and compensated cirrhosis who presented at hepatology clinics in tertiary referral centres (*n* = 299)	Biopsy‐defined	HCC was diagnosed by dynamic computed tomography scan, magnetic resonance imaging or biopsy HCC developed in 39 of 299 patients with MASH and compensated cirrhosis HCC development at 10 years, % (95% CI) With T2D:^c^ 25 (18–30) Without T2D:^c^ 7 (3–13) Annualised rates for HCC development, per 100 PYs (95% CI) With T2D:^c^ 3.1 (2.2–4.4) Without T2D:^c^ 1.2 (0.5–2.7)	There was a 4.2‐fold (95% CI: 1.2–14.2) increase in risk of developing HCC in patients with T2D than without T2D remained a significant predictor of HCC when analysed as a time‐dependent variable (adjusted sHR [95% CI]: 5.42 [1.74–16.80]; *p* = 0.003) Metformin use over time was associated with a reduced risk of HCC (adjusted sHR [95% CI]: 0.97 [0.95–0.99]; ref: no metformin use) in patients with HbA1c levels > 7%	Vilar‐Gomez et al. [72]

Abbreviations: AC, alcohol‐related cirrhosis; aHR, adjusted hazard ratio; AIH, autoimmune hepatitis cirrhosis; ALT, alanine transaminase; AST, aspartate transaminase; BMI, body mass index; CC, cryptogenic cirrhosis; CI, confidence interval; CTP, Child–Turcotte–Pugh; eGFR, estimated glomerular filtration rate; FIB‐4, fibrosis‐4 index; HbA1c, glycated haemoglobin; HBV, hepatitis B virus; HCC, hepatocellular carcinoma; HCV, hepatitis C virus; HR, hazard ratio; ICD‐9/10, International Classification of Diseases, Ninth/Tenth Revision; INR, international normalised ratio; IQR, interquartile range; KPS, Karnofsky Performance Scale; LRE, liver‐related event; LSM, liver stiffness measurement; MASH, metabolic dysfunction‐associated steatohepatitis; MASLD metabolic dysfunction‐associated steatotic liver disease; MELD, Model for End‐Stage Liver Disease; NASH, non‐alcoholic steatohepatitis; NCT, National Clinical Trial; NR, not reported; PY, person‐year; RCT, randomised controlled trial; ref, reference; SHR, subdistribution hazard ratio; sHR, sub‐hazard ratio; T2D, type 2 diabetes; UNOS, United Network for Organ Sharing; VA, Veteran Affairs; VCTE, vibration‐controlled transient elastography.

^a^ Factors reported as statistically significant (*p* < 0.05).

^b^ After controlling for cause of cirrhosis (MASH, CC, AC and AIH).

^c^ At baseline.

^d^ Adjusted by centre, calendar year of patients' recruitment, age, sex, race/ethnicity, BMI, smoking status, alcohol consumption, CTP score, oesophageal varices and antidiabetic medications at baseline.

^e^ Patients were defined as having MASLD if they had two or more elevated ALT values (≥ 40 IU/mL for men and ≥ 31 IU/mL for women) in the ambulatory setting and more than 6 months apart, with no positive serologic laboratory testing for HBV or HCV.

^f^ Such as ascites, encephalopathy, varices with or without bleeding.

^g^ Ascites, varices, thrombocytopenia, splenomegaly or hepatic encephalopathy.

**TABLE 3:** Performance of NITs for predicting liver‐related events, particularly decompensation in patients with cirrhosis due to MASH.

Study	Are et al. [82]	Calzadilla‐Bertot et al. [24]	Loomba et al. [85]
Population	Patients with MASH and compensated cirrhosis and portal hypertension who were enrolled in a phase 2 RCT (NCT02462967)	Patients with MASH and cirrhosis with no previous episodes of clinical decompensation	Patients aged 18–65 years with MASH and cirrhosis who participated in a phase 2 (NCT03053063) or phase 3 RCT (NCT01672879)
Definition of LREs	LREs were defined as at least one of the following: Development or progression of gastroesophageal varices, new‐onset ascites, variceal haemorrhage and hepatic encephalopathy An increase in CTP score of 2 points from baseline An increase in MELD score to > 15	First event of hepatic decompensation, defined by the occurrence of any of the following: ascites (identified or confirmed by abdominal ultrasound), upper gastrointestinal bleeding secondary to portal hypertension (confirmed by endoscopy in the presence of gastroesophageal varices or portal hypertensive gastropathy) or hepatic encephalopathy (established by clinical parameters, neuropsychological tests or electroencephalogram)	LREs were defined as clinically apparent ascites requiring treatment, grade ≥ 2 hepatic encephalopathy according to the West Haven criteria requiring treatment, and portal hypertension‐related gastrointestinal bleeding, liver transplantation, qualification for transplant (MELD ≥ 15) or mortality
Period of prediction	By week 52 of the RCT	At 5 and 10 years from the day of liver biopsy for diagnosing cirrhosis	By week 48 for participants in the phase 2 RCT and 96 weeks in the phase 3 RCT (NCT01672879)
*Performance of NITs for predicting LREs*			
**NIT**	**AUROC (95% CI)**	**AUROC (95% CI)**	**C‐statistic (95% CI)**
ABIDE	—	Derivation/validation cohorts At 5 years: 0.80 (0.73–0.84)^a^/0.78 (0.74–0.81) At 10 years: 0.76 (0.70–0.82)/0.78 (0.71–0.83)	—
ALBI‐FIB‐4 score	—	Derivation/validation cohorts At 5 years: 0.73 (0.66–0.79)/0.72 (0.60–0.83) At 10 years: 0.72 (0.66–0.79)/0.71 (0.69–0.78)	—
APRI score	0.58 (0.47–0.67)	—	—
ELF score	0.67 (0.57–0.77)^b^	—	0.80 (0.71–0.89)
FIB‐4 score	0.58 (0.47–0.69)	Derivation/validation cohorts At 5 years: 0.74 (0.70–0.79)/0.74 (0.68–0.80) At 10 years: 0.70 (0.63–0.76)/0.72 (0.70–0.85)	0.76 (0.66–0.86)
NFS	0.65 (0.55–0.75)	Derivation/validation cohorts At 5 years: 0.72 (0.66–0.78)/0.73 (0.67–0.79) At 10 years: 0.71 (0.67–0.73)/0.70 (0.63–0.77)	0.70 (0.60–0.80)
MELD score	0.57 (0.46–0.68)	Derivation/validation cohorts At 5 years: 0.69 (0.66–0.75)/0.68 (0.62–0.80) At 10 years: 0.61 (0.54–0.69)/0.64 (0.57–0.71)	—
CTP score	0.53 (0.48–0.58)	Derivation/validation cohorts At 5 years: 0.72 (0.64–0.79)/0.67 (0.63–0.75) At 10 years: 0.69 (0.63–0.75)/0.65 (0.62–0.69)	—
VCTE	—	—	0.77 (0.67–0.87)^c^

Abbreviations: ALBI, albumin‐bilirubin index; APRI, aspartate transaminase to platelet ratio index; AUROC, area under receiver operator characteristic curve; C, concordance; CI, confidence interval; CTP, Child–Turcotte–Pugh; ELF, Enhanced Liver Fibrosis; FIB‐4, fibrosis‐4 index; LRE, liver‐related event; LSM, liver stiffness measurement; MASH, metabolic‐dysfunction associated steatohepatitis; MELD, Model for End‐Stage Liver Disease; NCT, National Clinical Trial; NFS, non‐alcoholic fatty liver disease fibrosis score; NIT, non‐invasive test; NPV, negative predictive value; PPV, positive predictive value; RCT, randomised controlled trial; VCTE, vibration‐controlled transient elastography.

^a^ Optimal ABIDE cut‐off point was ≥ 4.1, with sensitivity of 79% and specificity of 85% for predicting LREs.

^b^ Optimal ELF threshold score was 9.8, with sensitivity of 87.9%, specificity of 26.6%, PPV of 23.6% and NPV of 89.5% for predicting LREs.

^c^ Optimal LSM by VCTE threshold was ≥ 30.7 kPa, sensitivity of 70%, specificity of 79%, PPV of 11% and NPV of 99% for predicting LREs.

There were very limited data on improvements in biopsy‐based outcomes in patients with cirrhosis due to MASH (see Data S2) [37, 38, 87]. Forty‐four studies reported data on the use of NITs to identify or diagnose cirrhosis in patients with MASH [18, 21, 22, 23, 25, 26, 29, 34, 35, 36, 39, 42, 43, 45, 47, 48, 50, 53, 54, 57, 60, 63, 64, 65, 66, 67, 69, 70, 71, 72, 73, 74, 75, 76, 77, 78, 79, 83, 84, 86, 87, 88, 89, 90]. Data from these studies are summarised in Data S2 and Table S7.

### 3.4 Clinical Outcomes Without Liver Transplant in Patients With Cirrhosis Due to MASH

#### 3.4.1 Survival Outcomes Without Liver Transplant

Three studies reported data on survival or death without liver transplant [65, 69, 72]. Although clinical setting and outcome definitions varied among studies (Table 2), survival rates were generally low and influenced by multiple factors [65, 69, 72].

In 2021, Tan et al. reported that patients with cirrhosis due to MASH (*n* = 99) who were hospitalised in Singapore for the management of first onset ascites had a transplant‐free survival rate of 27.2% at 60 months [65]. Cirrhosis was diagnosed based on a combination of biochemical, imaging and endoscopic features [65]. Independent factors found to predict cirrhosis‐related deaths or liver transplant in multivariable analysis were estimated glomerular filtration rate (eGFR) of < 60 mL/min/1.73 m^2^ (subdistribution hazard ratio [SHR]: 2.0) and Model for End‐Stage Liver Disease (MELD) score ≥ 15 (SHR: 4.2) [65].

A multinational study by Vilar‐Gomez et al. of 299 patients with MASH and compensated cirrhosis compared survival in patients with and without type 2 diabetes (T2D) [72]. In this retrospective study, cirrhosis was defined by biopsy and graded as Child–Turcotte–Pugh (CTP) A. There were 33 all‐cause deaths in the overall population and 10‐year transplant‐free survival was significantly lower in those with T2D (38%; 95% confidence interval [CI]: 31–45) than those without (81%; 95% CI: 75–77) [72]. Metformin use (as a time‐dependent variable) was associated independently with a reduced risk of all‐cause mortality (hazard ratio [HR]: 0.41; 95% CI: 0.26–0.45). Vilar‐Gomez et al. also reported data on decompensation and the development of HCC in this study population [72], which are presented in this report.

Thuluvath et al. estimated that the 5‐year cumulative incidence of all‐cause death or deterioration in patients with cirrhosis due to MASH (mean age 58 years) who remained on a liver transplant waiting list (United Network for Organ Sharing) was 35% in the period 2002 to 2016 in the USA (Table 2) [69]. A multivariable analysis revealed that cirrhosis due to MASH was associated with a higher risk of waiting list death or deterioration than cirrhosis due to alcohol‐related liver disease (ALD) or autoimmune hepatitis (AIH); the adjusted HR (aHR) (95% CI) was 0.92 (0.87–0.97) for ALD and 0.88 (0.80–0.96) for AIH [69]. After controlling for cirrhosis cause, factors that increased the risk of waiting list death or deterioration included poor performance status (Karnofsky Performance Scale score ≤ 30%; aHR: 1.78 [reference: ≥ 40%]), encephalopathy (aHR: 1.21), T2D (aHR: 1.11), high MELD score (aHR: 1.10 [reference unspecified]), Hispanic ethnicity (aHR: 1.13 [reference: White ethnicity]) and low serum albumin (aHR: 0.97 [reference unspecified]; Table 2) [69].

#### 3.4.2 Decompensation in Patients With Cirrhosis Due to MASH

Four studies reported data on decompensation in patients with MASH due to cirrhosis. In these studies, decompensation events included ascites, variceal or gastrointestinal bleeding, and encephalopathy. However, definitions of specific decompensation events, particularly ascites, varied among the studies (Table 2) [24, 72, 82, 85].

Vilar‐Gomez et al. conducted a multinational retrospective study of patients with compensated cirrhosis due to MASH in the period 1995 to 2016 (Table 2) [72]. The following were considered liver decompensation events: occurrence of ascites (identified clinically or by ultrasound), upper gastrointestinal bleeding secondary to portal hypertension (confirmed by endoscopy in the presence of gastroesophageal varices or hypertensive gastropathy), and hepatic encephalopathy (established by clinical parameters, neuropsychological tests, or electroencephalogram) [72]. Of 299 patients, 60 developed ascites, 18 had variceal bleeding, and five developed encephalopathy as first events of decompensation, with a median follow‐up of 5.1 years [72].

The 10‐year adjusted cumulative incidence of hepatic decompensation was higher in patients with T2D at baseline (51%; 95% CI: 44–59) than in patients without (26%; 95% CI: 17–33) [72]. A multivariable analysis adjusted for variables, including demographics, clinical status, investigations and antidiabetic medications, showed that metformin (as a time‐dependent variable) was associated independently with a reduced risk of decompensation (adjusted sub‐hazard ratio [sHR]: 0.80; 95% CI: 0.74–0.97) [72]. Metformin use at baseline lowered the risk of decompensation in patients with a baseline glycated haemoglobin (HbA1c) of > 7% (adjusted sHR: 0.97; 95% CI: 0.95–0.99) but not those with HbA1c of ≤ 7% (adjusted sHR: 1.00; 95% CI: 0.94–1.06) [72].

Three studies investigated the performance of different NITs for predicting liver‐related outcomes [24, 82, 85], including decompensation events, mortality, and liver transplant [82, 85]; however, only one study compared NITs for the prediction of decompensation events alone [24].

In an analysis of data from a phase 2 RCT that enrolled 162 patients with MASH and compensated cirrhosis in the USA, Are et al. defined liver‐related events as follows: development or progression of gastroesophageal varices, first onset ascites, variceal haemorrhage, hepatic encephalopathy, an increase in CTP score of 2 points from baseline, and an increase in MELD score to > 15 (Table 2) [82]. By week 52, 33 patients had developed at least one liver‐related event; the initial event was varices or progression of varices in 17 patients, an increased CTP score in 13 patients, and an increase in MELD score to > 15 in three patients [82].

The Enhanced Liver Fibrosis (ELF) test, which measures three direct markers of liver fibrosis (hyaluronic acid, type III procollagen peptide and tissue inhibitor of matrix metalloproteinase 1), and other NITs were evaluated as predictors of liver‐related events at 52 weeks. The area under the receiver operator characteristic curve (AUROC [95% CI]) for the ELF test was slightly greater than other NITs (AUROC [95% CI]: ELF, 0.67 [0.57–0.77]; non‐alcoholic fatty liver disease fibrosis score (NFS), 0.65 [0.55–0.75]; fibrosis‐4 index (FIB‐4), 0.58 [0.47–0.69]; aspartate transaminase to platelet ratio index (APRI), 0.58 [0.47–0.67]; MELD score, 0.57 [0.46–0.68]; CTP score, 0.53 [0.48–0.58], Table 3) [82]. An ELF score of > 9.8 was reported to have sensitivity, specificity, positive predictive value (PPV) and negative predictive value (NPV) of 87.9%, 26.6%, 23.6% and 89.5%, respectively, for predicting the liver‐related events at 52 weeks [82]. However, the AUROCs reported in the analysis suggested that NITs had poor‐to‐moderate predictive ability for the liver‐related outcomes [82].

Loomba et al. analysed RCT data from 734 patients with MASH and compensated cirrhosis [85]. Liver‐related events were defined as clinically apparent ascites requiring treatment, grade ≥ 2 hepatic encephalopathy requiring treatment, gastrointestinal bleeding related to portal hypertension, liver transplant, MELD score ≥ 15 and mortality [85]. During a median follow‐up of 16.2 months, 4% of patients had developed ascites, hepatic encephalopathy, gastrointestinal bleeding, qualified for liver transplant or had a liver transplant, or died (Table 2) [85]. This analysis showed that a baseline liver stiffness measurement (LSM) by vibration‐controlled transient elastography (VCTE) threshold of ≥ 30.7 kPa was optimal (concordance [C]‐statistic [95% CI]: 0.77 [0.67–0.87]) for predicting an approximately 10‐fold risk of liver‐related events. Baseline NFS, ELF and FIB‐4 performed similarly (C‐statistic [95% CI]: NFS, 0.70 [0.60–0.80]; ELF, 0.80 [0.71–0.89]; FIB‐4, 0.76 [0.66–0.86]; Table 3). The sensitivity, specificity, PPV and NPV of this LSM threshold (≥ 30.7 kPa) for predicting liver‐related events were 70%, 79%, 11% and 99%, respectively [85]. The LSM by VCTE threshold remained an independent predictor of liver‐related events after adjustment for age, sex, ethnicity and body mass index (aHR [95% CI]: 10.13 [4.38–23.41]) [85].

Agile 4 is a scoring system that combines LSM by VCTE with clinical characteristics and laboratory markers including platelets, aspartate transaminase (AST) and alanine transaminase (ALT) [85]. In patients with sufficient data for the analyses of Agile 4 (*n* = 701), the performance of LSM by VCTE and Agile 4 for predicting liver‐related events was similar (C‐statistic [95% CI]: LSM by VCTE, 0.81 [0.72–0.90], Agile 4, 0.82 [0.74–0.90]), suggesting that the additional parameters did not improve the prognostic utility of LSM by VCTE [85].

Calzadilla‐Bertot et al. developed a predictive model of liver decompensation in a multi‐country study of patients with cirrhosis due to MASH [24]. The primary study outcome was the first event of decompensation, defined by occurrence of ascites (identified or confirmed by ultrasound), upper gastrointestinal bleeding secondary to portal hypertension, or hepatic encephalopathy. The model (named ABIDE), comprising AST/ALT ratio, bilirubin levels, international normalised ratio (INR), the presence of T2D, and the presence of oesophageal varices [24], was derived using data from a cohort of 299 patients from Australia, Cuba, Hong Kong, and Spain, and validated in a cohort of 244 patients in the USA [24].

Independent predictors of decompensation in a multivariable analysis of data from the derivation cohort were the presence of oesophageal varices (sHR [95% CI]: 2.08 [1.16–3.61]) or T2D (2.25 [1.20–3.58]), raised AST/ALT ratio (2.28 [1.50–3.45]; reference unspecified), increased INR (2.28 [1.50–3.45]; reference unspecified) and elevated total bilirubin level (1.32 [1.14–1.67]; reference unspecified; Table 2) [24]. In the derivation cohort, the AUROC (95% CI) of ABIDE for predicting decompensation was 0.80 (0.73–0.84) at 5 years and similar at 10 years (0.76 [0.70–0.82]; Table 3). The accuracy (based on AUROC) of ABIDE for predicting decompensation at 5 and 10 years was significantly better (*p* < 0.05) for ABIDE than albumin‐bilirubin index (ALBI), MELD score, NFS, CTP score and ALBI‐FIB‐4 score in both derivation and validation cohorts (Table 3) [24].

#### 3.4.3 Development of HCC

Three studies reported data on the incidence and risk of developing HCC in patients with cirrhosis due to MASH [26, 54, 72]. The definition of cirrhosis varied among studies and was either based on biopsies or the presence of complications, including ascites, varices, and/or encephalopathy (Table 2) [26, 54, 72].

Pinyopornpanish et al. reported that in the period 2002 to 2016, HCC developed in 82 out of 950 patients with MASH stage F4 (cirrhosis) in the USA during a 4326‐person‐years (PYs) follow‐up, which ran from the date of diagnosis until the date of last abdominal imaging, liver transplant or HCC diagnosis (Table 2) [54]. The annual incidence of HCC was 1.90 per 100 PYs [54]. Multivariable analysis in patients with cirrhosis demonstrated that HCC development was associated with male sex (HR: 4.06), older age (HR: 1.05 [reference unspecified]) and a high CTP score (HR: 1.38 [reference unspecified]; Table 2). Statin use, however, was associated with a lower risk of developing HCC (HR: 0.40) [54].

In a US‐based registry study, Cholankeril et al. reported that 3013 patients with MASLD in the period 2004 to 2008 were identified as having cirrhosis (based on International Classification of Diseases [ICD] Ninth/Tenth Revision codes), of which 690 patients had FIB‐4 scores of > 2.67 at baseline and 3 years after diagnosis of cirrhosis (Table 2) [26]. This group of patients had the highest incidence of HCC (2.66 per 100 PYs). The incidence of HCC was lowest in patients with cirrhosis who had FIB‐4 scores of < 1.45 over the same 3‐year period, at 3.43 per 100 PYs (95% CI: 1.88–5.76) [26].

Vilar‐Gomez et al. reported that the 10‐year risk of HCC development in MASH was significantly higher in patients with T2D than those without T2D (25% vs. 7%; *p* < 0.01; Table 2) [72]. In their retrospective multinational study of 299 patients with cirrhosis due to MASH in the period 1995 to 2016, the annualised rates per 100 PYs for developing HCC were 3.1 and 1.2 in patients with T2D and without T2D, respectively. In a multivariable analysis, a 4.2‐fold (95% CI: 1.2–14.2) increased risk of HCC was observed in patients with T2D compared with those without T2D. T2D remained a significant predictor of HCC when analysed as a time‐dependent variable (adjusted sHR [95% CI]: 5.42 [1.74–16.80]). Notably, metformin use over time was associated independently with a reduced risk of developing HCC (sHR [95% CI]: 0.78 [0.69–0.96]). However, the associated benefit of metformin use was seen only in patients with HbA1c levels of > 7% (adjusted sHR [95% CI]: 0.67 [0.45–0.84]).

### 3.5 Clinical Outcomes After Liver Transplant in Patients With Cirrhosis Due to MASH

Seven studies estimated post‐liver transplant survival in patients with MASH stage F4 (cirrhosis), with 5‐year survival frequently being reported as ≥ 70% across different countries (Table S6) [17, 28, 30, 31, 41, 59, 73]. Three studies reported data from the USA, with 5‐year survival ranging from 76% to 83% [17, 28, 59]. Similar results were reported in Austria [41], France [73], Iran [30], Italy [31] and Switzerland [73], with survival rates in these countries ranging from 72% to 85% at 5 years (Table S6).

Cardiovascular disease or cardiovascular events, infection and sepsis were the main causes of death after liver transplant in patients with cirrhosis due to MASH in the studies identified [49, 73]. In a study of 361 patients in France and Switzerland who underwent liver transplant between 2000 and 2019, there were 72 deaths after transplant with a median time to death of 11.5 months [73]. Four of nine deaths (44.4%) in the first 30 days after transplant were of cardiovascular origin. Over a 10‐year post‐transplant period, the main causes of death were infectious disease (37.5%), malignancy (29.2%) and cardiovascular events (22.2%), respectively [73]. In the USA, the leading causes of mortality 1 year after liver transplant for MASH were infection (16.1%) and cardiovascular disease (11.5%) [49].

A multivariable analysis of registry data from 6344 patients in the USA who underwent liver transplant for MASH during 2008 to 2017 showed that the independent risk factors for overall all‐cause mortality after liver transplant included diabetes (HR: 1.14), HCC (HR: 1.25), low Karnofsky Performance Scale score (10%–30%; HR: 1.70 [reference: 70%–100%]), life support requirement (HR: 1.23), grade III or IV encephalopathy (HR: 1.31), retransplant (HR: 1.75), and receiving a liver from a Hispanic donor (HR: 1.20 [reference: White donors]) or liver donated after cardiac death (HR: 1.47 [reference: livers from living donors]) [49]. In a study in Austria examining 65 liver transplants for MASH with donors who were deceased (2002–2012), recipient survival was poorer with livers from older donors (> 55 years vs. ≤ 55 years; *p* = 0.013); however, survival rates were not reported [41]. In other studies, patients with poor functional status who required total assistance [44] and those with comorbid coronary artery disease [58] were found to be at increased risk of overall post‐liver transplant mortality.

Few data were identified on the recurrence of cirrhosis after liver transplant. In an observational study of 150 patients with cirrhosis due to MASLD who underwent liver transplant in France or Switzerland in the period 2000 to 2019, repeated liver graft biopsies revealed that cirrhosis related to MASLD recurred after a median of 7.1 years in 4% of patients [74]. Risk factors for the recurrence of advanced fibrosis (stages F3 and F4) identified in multivariable analysis were metabolic syndrome before liver transplant (odds ratio [OR]: 8.6), long‐term use of cyclosporine A (OR: 11.4), and grade 2 steatosis 1 year after liver transplant (OR: 10.7 [reference unspecified]). Initial graft steatosis and donor or recipient age did not affect the occurrence of advanced fibrosis.

Limited data from three studies that reported data on patient and donor characteristics linked with transplant graft failure are summarised in Data S2.

## 4 Discussion

This SLR identified data from both observational and interventional studies published over a 10‐year period (2014–2024) for a range of outcomes in patients with cirrhosis due to MASH in transplant and non‐transplant settings. Most studies identified were observational in design and their duration ranged widely (2–31 years). In contrast, the few RCTs in this SLR generally had a duration of 2–3 years. Here we have synthesised data from studies on key clinical outcomes related to cirrhosis due to MASH. For outcomes in patients without liver transplant, there were limited data on survival and liver‐related events, including decompensation and HCC, with three or four studies for each of these outcomes. The heterogeneity of these studies precluded aggregation or quantitative synthesis of the data.

There were limited data on survival outcomes without liver transplant. In one study, the 5‐year transplant‐free survival was estimated as 27.2% in patients with MASH who were hospitalised with decompensated cirrhosis; low eGFR (< 60 mL/min/1.73 m^2^) and MELD score ≥ 15 were associated with reduced transplant‐free survival [65]. In patients with MASH and compensated cirrhosis, 10‐year transplant‐free survival was lower in patients with T2D than without (38% vs. 81%) [72]. Cirrhosis due to MASH was associated with a higher risk of waiting list death or deterioration compared with cirrhosis from other causes in the period 2002 to 2016 in the USA [69], which remained the case in a later study of patients on the transplant waitlist in 2016 to 2021, which was not captured in our SLR [91]. Survival in patients with MASH improved after liver transplant and was reported as ≥ 70% at 5 years across different countries [17, 28, 30, 31, 41, 59, 73]. However, it is difficult to ascertain the impact of MASH per se on post‐transplant survival because the intervention is influenced by the complexity of pre‐ and post‐operative factors that are often independent of the baseline liver disease [92].

Two studies reported limited data on decompensation events, likely from the same cohort of patients with MASH and cirrhosis, in the period 1995–2016 [24, 72]. Decompensation events in these studies were ascites, gastrointestinal bleeding secondary to portal hypertension and encephalopathy [24, 72]. These events occurred in 28% of patients with a median follow‐up of 5.1 years [72]. Ascites and variceal bleeding were the most common first events of decompensation [72]. Predictors of decompensation were the presence of oesophageal varices, T2D, increased INR, raised total bilirubin level, and increased AST/ALT ratio [72].

Three studies compared NITs for the prediction of decompensation events or liver‐related events that were a composite of outcomes including decompensation, mortality, and liver transplant [24, 82, 85]. Although ascites, gastrointestinal bleeding secondary to portal hypertension and encephalopathy were considered decompensation events in these studies, the definitions of these events differed among these studies. For example, ascites was defined as clinically apparent and requiring treatment in one study [85], but needed to be identified by abdominal ultrasound only in another study [24]. One study developed and validated ABIDE for predicting decompensation events [24]. Compared with the ALBI, MELD score, NFS, CTP score and ALBI‐FIB‐4 score, ABIDE was reported to be more accurate for predicting decompensation events specifically at 5 and 10 years, with AUROCs of 0.8 [24]. Other studies, not included in this SLR, have reported C‐statistics of > 0.8 for ANTICIPATE‐NASH models for predicting a composite of liver‐related events (including first decompensation, HCC, liver transplant and liver‐related death) at 3 years from baseline in patients with MASLD (all fibrosis stages, or stages F3 and F4 combined) [93, 94]. In a study published in 2025, ANTICIPATE‐NASH models were reported to be superior to liver histology for predicting liver‐related events at 3 years in patients with advanced fibrosis (stages F3 and F4) due to MASH [93].

This SLR identified three studies that investigated risk factors for HCC in patients with cirrhosis due to MASH [26, 54, 72]. Definitions of cirrhosis varied among studies and relied on biopsies, symptoms or signs of portal hypertension, or radiological evidence [26, 54, 72]. In these patients, the development of HCC was identified based on ICD codes in a registry‐based study [26] or diagnosed based on imaging or biopsies in other studies [54, 72]. Increased age, male sex, the presence of T2D, and high CTP scores were associated with a higher risk of developing HCC, whereas statin or metformin use was associated with a lower risk of developing HCC [26, 72]. In a study of cirrhosis across different aetiologies that was not included in this SLR, older age and male sex were also associated with the development of HCC [95].

This SLR has identified several potential evidence gaps. Non‐fatal cardiovascular events in patients with cirrhosis due to MASH did not emerge as a theme in this SLR. However, previous studies not captured in this SLR have highlighted an increased risk of cardiac arrhythmias and major adverse cardiovascular events (including non‐fatal myocardial infarction, non‐fatal stroke and cardiovascular death) in patients with MASLD compared with population‐matched controls [96, 97, 98, 99]. In adults, these risks were evident across all stages of MASLD, and increased with disease severity [96, 99]. Furthermore, while survival in patients with cirrhosis due to MASH improves after liver transplant, cirrhosis can recur in these patients [74]. There is a clear need for early detection of cirrhosis in these patient groups. However, studies have relied on a diagnosis of cirrhosis based on biopsy, which is likely delayed relative to the onset of the condition. Furthermore, in our experience, liver biopsies are not performed per protocol after liver transplant in most centres and routine blood investigations often fail to detect early disease recurrence.

Prognostic prediction models can help to identify patients at high risk of liver‐related events who require early treatment [100]. These models can be employed to improve clinical trial efficiency by informing selection of suitable patients who are at risk for an outcome of interest, potentially shortening development timelines for new therapies for cirrhosis due to MASH [101]. However, only three studies comparing the performance of prognostic prediction models were identified in this SLR. The definitions used for cirrhosis and liver‐related outcomes, including decompensation events, varied among these studies, which precluded further data aggregation. Harmonisation of these definitions in future studies would help to improve comparability and generalisability, as has been attempted in MASLD research based on electronic health records [102].

The limitations of this SLR must be acknowledged. The focus of the SLR was cirrhosis due to MASH, and the electronic search terms did not include MASLD or non‐alcoholic fatty liver disease (NAFLD). As such, publications that employed those terms only in reference to cirrhosis may not have been identified in the searches. Due to the volume of studies identified in the electronic searches, we performed a single screening, which may have affected which studies were identified. Given the focus on MASH with cirrhosis, studies that reported data on advanced fibrosis (stages F3 and F4) but that did not report data on cirrhosis (stage F4) separately were excluded. In addition, studies reporting potentially relevant outcomes, but which did not include the search terms of interest in the title or abstract or did not indicate that data were reported for the specific population of interest, would have either been excluded during the electronic searches or considered ineligible in title/abstract screening. For example, portal hypertension was not listed as a search term or relevant clinical outcome; studies on the use of ANTICIPATE‐NASH models for identifying clinically significant portal hypertension defined by hepatic venous pressure gradient were not included by this SLR [103, 104]. Finally, the broad scope of this SLR and the heterogeneity of the few studies identified precluded quantitative data synthesis or aggregation, which may limit the interpretation and generalisability of the findings.

In conclusion, this SLR identified a broad range of insights on clinical outcomes in MASH with cirrhosis, as well as key data on survival without transplant, decompensation events, and the development of HCC. Our results suggest that some evidence gaps in the literature remain. In particular, there was very limited evidence for the role of prognostic prediction models for liver‐related events in this patient population. Given that potential treatments for cirrhosis due to MASH are in development, further studies are required to address the evidence gaps identified.

## Author Contributions

C.H., R.O., and F.T. designed the systematic review. All authors were involved in drafting the article or revising it critically for important intellectual content, and all authors approved the final version to be submitted for publication.

## Funding

Oxford PharmaGenesis was funded by Novo Nordisk A/S to undertake this systematic literature review. Medical writing support was provided by Mike Lee, PhD of Oxford PharmaGenesis, Oxford, UK and was funded by Novo Nordisk A/S in accordance with Good Publication Practice (GPP 2022) guidelines (www.ismpp.org/gpp‐2022).

## Ethics Statement

The authors have nothing to report.

## Conflicts of Interest

H.H.'s institutions have received research funding from AstraZeneca, Echosens, Gilead, Intercept, MSD, Novo Nordisk, Pfizer and Takeda. He has served as a consultant, speaker and on advisory boards for AstraZeneca, Boehringer Ingelheim, Bristol Myers Squibb, Echosens, GSK, Ipsen, MSD and Novo Nordisk, and has been part of hepatic events adjudication committees for Arrowhead, Boehringer Ingelheim, GW Pharma and KOWA. C.H. is an employee of Oxford PharmaGenesis. Oxford PharmaGenesis received funding from Novo Nordisk A/S to conduct this SLR. J.V.L. has received grants to his institutions from Boehringer Ingelheim, Echosens, Gilead Sciences, Madrigal Pharmaceuticals, MSD, Novo Nordisk, Pfizer and Roche Diagnostics; consulting fees from Echosens, The Global NASH/MASH Council, GSK, Madrigal Pharmaceuticals, Novo Nordisk and Pfizer; and fees for lectures from Echosens, Gilead Sciences, GSK, MSD, Novo Nordisk, Pfizer and ProSciento outside of the submitted work. R.O. was an employee and shareholder of Novo Nordisk A/S at the time of the study. M.E.R. has acted as a scientific consultant for 89bio, Akero, Boehringer Ingelheim, Eli Lilly, GSK, Hepta Bio, Histoindex, Inventiva, Madrigal Pharmaceuticals, Novo Nordisk, Regeneron and Sagimet Biosciences. G.S. has acted as a speaker for AbbVie, Eli Lilly, Gilead, Merck and Novo Nordisk and has served as an advisory board member for Gilead, GSK, Merck and Novo Nordisk. F.T. was an employee of Novo Nordisk A/S at the time of the study. Z.M.Y. has received research funding and/or served as consultant/advisor to Abbott, Akero Therapeutics, Aligos Therapeutics, Boehringer Ingelheim, Bristol Myers Squibb, CymaBay, GSK, Intercept, Ipsen, Madrigal Pharmaceuticals, Merck, Novo Nordisk, Sanofi and Siemens. M.N. consults for and has received grants from Gilead, GSK, Madrigal Pharmaceuticals, Novo Nordisk, Takeda and Terns. He consults for, advises, and owns stock in CytoDyn. He consults for and advises 89bio, Altimmune, Boehringer Ingelheim, Merck and Perspectum. He has received grants from and owns stock in Viking Therapeutics. He advises Blade Therapeutics, cohBar, EchoSens, Fractyl Health, Intercept, NorthSea Therapeutics, Pfizer, Siemens and Roche Diagnostics. He has received grants from Allergan, Bristol Myers Squibb, Conatus Therapeutics, Enanta, Galectin Therapeutics, Galmed Pharmaceuticals, Genfit, Madrigal Pharmaceuticals, Novartis, Pfizer, Shire and Zydus. He owns stock in Anaetos, ChronWell, Cima and Rivus Pharmaceuticals.

## Supporting information


**Data S1:** liv70608‐sup‐0001‐DataS1.docx.

**Data S2:** liv70608‐sup‐0002‐Supinfo.docx.

## Data Availability

The data that support the findings of this study are derived entirely from publicly available sources. These data were accessed from Embase, MEDLINE and the Cochrane Library, and are cited in the reference section of this manuscript. No new data were generated or analysed specifically for this study. All relevant data are included within the article and its Data S2.
