# Supplementary material for: Clinical Outcomes and Non‐Invasive Testing in Metabolic Dysfunction‐Associated Steatohepatitis With Cirrhosis: A Systematic Review
Source: Liver Int. 2026 Mar 28;46(5):e70608. doi: 10.1111/liv.70608 (PMC13032177; doi:10.1111/liv.70608)
Supplement: Supplementary file 2 — Data S2: liv70608‐sup‐0002‐Supinfo.docx. [file LIV-46-0-s002.docx]

# Supporting Information for:

## Clinical Outcomes and Non-Invasive Testing in Metabolic Dysfunction-Associated Steatohepatitis With Cirrhosis: A Systematic Review

Authors: Hannes Hagström, Chris Hellmund, Jeffrey V Lazarus, Riku Ota, Mary E Rinella, Giada Sebastiani, Fotis Tefos, Zobair M Younossi, Mazen Noureddin

| **Contents** | **Page** |
| --- | --- |
| **Supplemental Results** |  |
| Factors Predicting Graft Failure After Liver Transplant | 3 |
| Non-Invasive Identification of Cirrhosis in Patients with MASH | 3 |
| Improvements in Biopsy-Based Outcomes in Patients with Cirrhosis due to MASH | 6 |
| **Supplemental Tables** |  |
| **Table S1**. Electronic search strings for Embase | 7 |
| **Table S2**. Electronic search strings for Ovid MEDLINE and Epub Ahead of Print, In-Process, In-Data-Review and Other Non-Indexed Citations, Daily and Versions | 9 |
| **Table S3**. Electronic search strings for Cochrane (Cochrane Database of Systematic Review [CDSR], Database of Abstracts of Reviews of Effects [DARE], Cochrane Central Register of Controlled trials [CENTRAL], Cochrane Methodology Register [CMR], National Health Service Economic Evaluation Database [NHS EED], Health Technology Assessment [HTA] Database and the American College of Physicians [ACP] journal club) | 11 |
| **Table S4**. List of eligible studies after full-text review (*n* = 317) grouped by inclusion and exclusion from data extraction | 13 |
| **Table S5**. List of 74 eligible studies with data extracted for clinical outcomes and use of NITs | 46 |
| **Table S6**. Post-liver transplant survival in MASH stage F4 | 58 |
| **Table S7**. Studies reporting AUROCs of NITs for identifying cirrhosis in patients with MASH | 59 |
| **References** | 61 |

# Supplemental Results

### Factors Predicting Graft Failure After Liver Transplant

Several patient and donor characteristics were linked with transplant graft failure in the studies identified in the systematic literature review (SLR). In a multivariable analysis of registry data from 53,788 patients in the USA who underwent liver transplant (including 5258 patients with metabolic dysfunction-associated steatohepatitis [MASH]), independent predictors of graft failure in all patients over the first 3 years after liver transplant included increasing recipient age in years (hazard ratio [HR]: 1.01 [reference unspecified]), presence of diabetes (HR: 1.19), increased Model for End-Stage Liver Disease (MELD) score (HR: 1.02 [reference unspecified]), hepatocellular carcinoma (HCC) exception (HR: 1.18), and need for life support (HR: 1.64) [[1](#_ENREF_1)]. Poorer graft survival was also found after transplant with livers from older donors (> 55 years) in the study in Austria mentioned previously [[2](#_ENREF_2)]. In 176 patients in Canada, graft survival was significantly poorer in patients with underweight or healthy weight than in those with overweight or obesity (78.6% vs. 98.6% at 1 year, *p* = 0.002) [[3](#_ENREF_3)].

## Non-Invasive Identification of Cirrhosis in Patients With MASH

A total of 44 studies reported data on the use of various non-invasive tests (NITs) for identifying cirrhosis in patients with MASH [[1](#_ENREF_1),[4-46](#_ENREF_4)]. A key theme that emerged was the use of fibrosis-4 index (FIB-4) and comparisons with other NITs, particularly vibration-controlled transient elastography (VCTE), to identify cirrhosis. FIB-4 is a blood-based NIT and comprises a score calculated based on age, aspartate transaminase (AST), alanine transaminase (ALT) and platelet count [[47](#_ENREF_47)], whereas VCTE is an imaging modality in which the velocity of low-frequency shear waves created by an ultrasound probe correlates to the stiffness or elasticity of the liver, resulting in a liver stiffness measurement (LSM), which is a marker of hepatic fibrosis [[39](#_ENREF_39)].

The use of FIB-4 was frequently reported in studies that evaluated the performance of NITs for identifying cirrhosis in patients with MASH (**Table S7**) [[17](#_ENREF_17),[25](#_ENREF_25),[33](#_ENREF_33),[34](#_ENREF_34),[39](#_ENREF_39),[45](#_ENREF_45),[46](#_ENREF_46)]. However, these studies differed in terms of study populations and the reference standards used for the diagnosis of cirrhosis.

In a study based on data in the National Health and Nutrition Examination Survey 2017–2020 in the USA, 6359 patients with metabolic dysfunction-associated steatotic liver disease (MASLD) were identified based on controlled attenuation parameter (CAP) ≥ 288 dB/m and at least one cardiometabolic risk factor [[33](#_ENREF_33)]. An LSM by VCTE ≥ 13.6 kPa was used as the reference standard for the diagnosis of cirrhosis in these patients. In this study, FIB-4 did not perform as well as the non-alcoholic fatty liver disease fibrosis score (NFS) and it performed similarly to the aspartate transaminase to platelet ratio index (APRI) for the detection of cirrhosis when applied to the entire study population. The area under the receiver operator characteristic curve (AUROC) (95% confidence interval [CI]) for NFS was greater than for FIB-4 and for APRI (NFS, 0.81 [0.77–0.85]; FIB-4, 0.67 [0.62–0.72]; APRI, 0.65 [0.60–0.70], **Table S7**) [[33](#_ENREF_33)]. Another study reported that FIB-4 performed similarly to the NFS, and both FIB-4 and NFS performed better than APRI and other NITs for detecting cirrhosis in MASLD. In this study, liver biopsy was used to identify cirrhosis due to MASLD/MASH in patient cohorts from the non-alcoholic steatohepatitis (NASH) Clinical Research Network (CRN) in the USA (*n* = 1483) and a specialist outpatient clinic in the UK (*n* = 494). Optimal cutoffs for seven NITs, including FIB-4, were derived from the NASH CRN cohort and validated in the UK cohort [[46](#_ENREF_46)]. Among the NITs evaluated, FIB-4 and the NFS had high AUROCs (> 0.80) to exclude cirrhosis (**Table S7**) [[46](#_ENREF_46)].

There was some evidence that LSM by VCTE performed better than blood-based NITs for identifying or diagnosing cirrhosis. An individual participant data meta-analysis of patients with MASLD was conducted to evaluate the performance of NITs with liver biopsy as the reference standard [[25](#_ENREF_25)]. This meta-analysis used data from studies in Europe, Asia and North America; most studies were cross-sectional where the study design was reported. Data from LSM by VCTE, FIB-4 and NFS in 2281 patients were used in AUROC curve analyses [[25](#_ENREF_25)]. LSM by VCTE at a threshold of 10 kPa had an AUROC (CI) of 0.84 (0.82–0.87) for identifying cirrhosis in patients with MASLD. Thresholds of 1.15 for FIB-4 and −1.866 for NFS performed similarly to 10 kPa for LSM by VCTE (AUROC [CI]: FIB-4, 0.81 [0.77–0.84]; NFS, 0.76 [0.72–0.79]; LSM by VCTE, 0.84 [0.82–0.87], **Table S6**); however, more patients would have needed liver biopsy if they were screened using FIB-4 or NFS [[25](#_ENREF_25)]. In another study of 1073 patients with MASLD in the USA, FibroMeter combined with VCTE had an AUROC (CI) of 0.90 (0.84–0.95) and was superior to FIB-4 (AUROC [CI]: 0.81 [0.77–0.85]) for identifying biopsy-defined cirrhosis [[45](#_ENREF_45)].

One study based in a hospital in Japan compared different methods of measuring liver stiffness [[17](#_ENREF_17)]. The study, which included 231 patients with MASLD, reported that LSM by VCTE was significantly less accurate for diagnosing biopsy-defined cirrhosis compared with LSM by magnetic resonance elastography (MRE) (AUROC [CI]: VCTE, 0.87 [0.81–0.92]; MRE, 0.92 [0.87–0.96]; *p* = 0.04) [[17](#_ENREF_17)].

Agile 4 and FibroScan-AST (FAST) are scoring systems that combined VCTE- and blood-based parameters [[39](#_ENREF_39)]. Both performed similarly to LSM by VCTE alone for diagnosing cirrhosis in patients with MASLD. The AUROC (CI) for Agile 4 and LSM by VCTE alone were similar at 0.85 (0.81–0.88) and 0.83 (0.78–0.87), respectively, in a study of 144 patients with biopsy-defined MASLD in the USA [[39](#_ENREF_39)]. However, the percentage of patients with indeterminate results was lower with Agile 4 than LSM by VCTE alone, suggesting that Agile 4 is more useful in clinical settings [[39](#_ENREF_39)]. In a meta-analysis of 2281 patients with MASH, the performance of FAST at a threshold of 0.47 was similar to LSM by VCTE at a threshold of 10 kPa for identifying cirrhosis (AUROC [CI]: FAST, 0.79 [0.76–0.82]; LSM by VCTE, 0.84 [0.82–0.87]) [[25](#_ENREF_25)].

One study reported data indicating that machine learning approaches may be superior to FIB-4 scoring for identifying cirrhosis in patients with MASLD. In 1370 patients with MASLD in the USA who underwent liver biopsy, a machine learning model (random forest [RF]) trained with 17 features (clinical and demographic), demonstrated greater accuracy and a larger AUROC for identifying clinically significant MASLD‐associated liver cirrhosis than FIB-4 or FibroScan (AUROC [CI]: RF, 0.89 [0.86–0.92]; FibroScan 0.86 [0.82–0.89]; FIB-4, 0.85 [0.81–0.88]) [[34](#_ENREF_34)]. However, the model was not validated in an independent cohort in this study [[34](#_ENREF_34)].

There were limited data (one study) on the use of sequential NIT strategies for identifying biopsy-defined cirrhosis in patients with MASH [[25](#_ENREF_25)]. The study examined the performance of the following sequences: FIB-4 followed by LSM by VCTE (FIB-4/LSM by VCTE), NFS followed by LSM by VCTE (NFS/LSM by VCTE), FIB-4 followed by FAST (FIB-4/FAST) and NFS followed by FAST (NFS/FAST). The thresholds used for each NIT (FIB-4: 0.7, LSM by VCTE: 16 kPa, NFS: −3.272, FAST: 0.35) were selected from the literature or previous analyses included in the study to minimise the screen failure rate (SFR) while maximising sensitivity. FIB-4/LSM by VCTE and NFS/LSM by VCTE performed similarly with a sensitivity of 59% and SFR of 76%, and identified four true-positive cases per 100 patients. Compared with FIB-4/LSM by VCTE and NFS/LSM by VCTE, FIB-4/FAST and NFS/FAST performed better with sensitivities of 91–92% and SFRs of 88–89%, and identified six true-positive cases per 100 cases [[25](#_ENREF_25)].

## **Improvements in Biopsy-Based Outcomes in Patients With Cirrhosis due to MASH**

A small number of studies reported improvements in biopsy-based outcomes in patients with cirrhosis due to MASH [[26](#_ENREF_26),[48](#_ENREF_48),[49](#_ENREF_49)]. These improvements were defined histologically as resolution of MASH, ≥ 1 stage improvement in fibrosis, and ≥ 2-point improvement in non-alcoholic fatty liver disease activity score (NAS). In a pooled analysis of placebo and treatment arms from two randomised controlled trials (RCTs) in patients with compensated cirrhosis, 16% of patients experienced a ≥ 1 stage improvement in fibrosis based on histology over the trial periods [[26](#_ENREF_26)]. As placebo and treatment arm data were pooled, it is unclear if the improvements observed were due to treatment or non-treatment effects. However, the authors justified this pooling because these RCTs were halted due to a lack of treatment efficacy [[26](#_ENREF_26),[48](#_ENREF_48),[49](#_ENREF_49)]. Compared with patients who did not improve, patients who did improve also showed corresponding lower risks of liver-related events and greater improvements in NIT scores and blood biomarker concentrations [[26](#_ENREF_26)]. There were some differences in the baseline characteristics of patients who improved compared with those who did not. At baseline, median body mass index was 32.4 kg/m^2^ (interquartile range, 27.7–37.2) in patients who improved and 33.3 kg/m^2^ (29.3–38.0) in those who did not improve; however, the proportion of patients with diabetes did not differ significantly between the groups (with improvement, 70%; without improvement, 76%; *p* = 0.11). Baseline scores of NITs including FIB-4, Enhanced Liver Fibrosis (ELF), NFS and LSM by VCTE, were significantly better (*p* < 0.0001) in patients who showed improved histological outcomes than those who did not [[26](#_ENREF_26)].

# Supplemental Tables

## Table S1. Electronic search strings for Embase

| # | Searches | Results |
| --- | --- | --- |
| 1 | exp nonalcoholic steatohepatitis/or (non-alcoholic steatohepatitis or nonalcoholic steatohepatitis or Metabolic dysfunction-associated steatohepatitis or NASH or MASH).mp. | 41025 |
| 2 | (F4 or cirrhosis).mp. | 259901 |
| 3 | ((Improvement adj5 fibrosis) or (cirrhosis adj5 regression) or alanine transaminase or ALT or aspartate transaminase or AST or decompens* or transplan* or hepatocellular carcinoma or HCC or ascites or haemorrhage or hemorrhage or encephalopathy or mortality or death).mp. | 4979130 |
| 4 | (NASH-CHECK or Short Form-36 or SF-36 or Short Form-6D or SF-6D or EQ-5D-5L or EQ-5D-5L-3L or EQ-5D or Chronic Liver Disease Questionnaire or CLDQ or Chronic Liver Disease Questionnaire-NASH or CLDQ-NASH or "Work Productivity and Activity Impairment" or WPAI or WPAI:SHP or activities of daily living or ADL or fatigue or pain or functional impairment or activity limitations or PROMIS-Fatigue).mp. | 2086166 |
| 5 | "health care cost"/or "drug cost"/or "hospital cost"/or "hospitalization cost"/or "nursing cost"/or ((health or global) adj2 burden).mp. or ((direct or indirect or societ* or employe*) adj2 (resource* or benefit*)).mp. or exp caregiver burden/or exp caregiver support/or (caregiver* or carer*).mp. or economics/or budget*.mp. or cost*.mp. or productivity/or productivity.mp. or absenteeism.mp. or absenteeism/or presenteeism.mp. or presenteeism/or "length of stay"/or Cost control/or (fiscal or financ* or funding).mp. or financial management.mp. or financial management/or health care utilization/or health care utili*.mp. or health care financing.mp. or health care financing/or health economics.mp. or health economics/or (burden adj2 (illness or disease$ or treatment*)).mp. or resource allocation/or budget/or pharmacoeconomics/or pharmacoeconomic*.mp. or pay?r.mp. or health care planning.mp. or health care planning/or (resource adj2 (use* or utili?ation or allocat* or burden or health)).mp. or (economic adj5 (burden or impact)).mp. or cost of illness.mp. or "cost of illness"/or cost control.mp. or "cost control"/or Economics, Medical/ | 2772652 |
| 6 | ("enhanced liver fibrosis" or ELF or FibroScan or FAST or "vibration-controlled transient elastography" or VCTE or "Fibrosis-4" or FIB-4 or "model for end-stage liver disease" or MELD or "NAFLD Activity Score" or NAS or non-invasive test*).mp. | 508403 |
| 7 | or/3-6 | 9279798 |
| 8 | Clinical Trial/ | 1088727 |
| 9 | Randomized Controlled Trial/ | 836064 |
| 10 | controlled clinical trial/ | 473635 |
| 11 | multicenter study/ | 400341 |
| 12 | Phase 1 clinical trial/ | 77368 |
| 13 | Phase 2 clinical trial/ | 115604 |
| 14 | Phase 3 clinical trial/ | 77021 |
| 15 | Phase 4 clinical trial/ | 7493 |
| 16 | exp RANDOMIZATION/ | 100163 |
| 17 | Single Blind Procedure/ | 55795 |
| 18 | Double Blind Procedure/ | 222064 |
| 19 | Crossover Procedure/ | 79068 |
| 20 | PLACEBO/ | 416062 |
| 21 | randomi?ed controlled trial$.tw. | 354509 |
| 22 | rct.tw. | 58885 |
| 23 | (random$ adj2 allocat$).tw. | 58246 |
| 24 | single blind$.tw. | 33665 |
| 25 | double blind$.tw. | 254587 |
| 26 | ((treble or triple) adj blind$).tw. | 2185 |
| 27 | placebo$.tw. | 383522 |
| 28 | Prospective Study/ | 932852 |
| 29 | (single arm or single-arm or noncomparative or non-comparative).tw. | 39549 |
| 30 | (Nonrandom* or non random* or non-random* or quasi-random* or quasirandom* or (phase adj3 (study or studies or trial*)) or ((crossover or cross-over) adj3 (study or studies or trial*)) or ((multicent* or multi-cent*) adj3 (study or studies or trial*))).ti,ab,hw,kf. or allocated.ti,ab,hw. or ((open label or open-label) adj5 (study or studies or trial*)).ti,ab,hw,kf. or ((equivalence or superiority or non-inferiority or noninferiority) adj3 (study or studies or trial*)).ti,ab,hw,kf. or (pragmatic study or pragmatic studies).ti,ab,hw,kf. or ((pragmatic or practical) adj3 trial*).ti,ab,hw,kf. or ((quasiexperimental or quasi-experimental) adj3 (study or studies or trial*)).ti,ab,hw,kf. or trial.ti,kf. | 1501098 |
| 31 | Clinical study/or Case control study/or Family study/or Longitudinal study/or Retrospective study/or (Prospective study/not Randomized controlled trials/) or Cohort analysis/or (Cohort adj (study or studies)).mp. or (Case control adj (study or studies)).tw. or (follow up adj (study or studies)).tw. or (observational adj (study or studies)).tw. or (epidemiologic$ adj (study or studies)).tw. or (cross sectional adj (study or studies)).tw. or (registry or register$ or survey).ti,ab. or (real world or RWE).ti,ab. or Real-life.ti,ab. or exp seroepidemiologic studies/or (descriptive adj3 (study or studies or design or analysis or analyses)).ti,ab,kf. or ((multidimensional or (multi adj dimensional)) adj3 (study or studies or design or analysis or analyses)).ti,ab,kf. | 5703940 |
| 32 | or/8-31 | 7806978 |
| 33 | Case study/ | 102377 |
| 34 | Case report.tw. | 579250 |
| 35 | Letter/ | 1249941 |
| 36 | or/33-35 | 1915222 |
| 37 | 32 not 36 | 7643850 |
| 38 | 1 and 2 and 7 and 37 | 4469 |
| 39 | (animal$ not human$).sh,hw. | 4992047 |
| 40 | 38 not 39 | 4418 |
| 41 | limit 40 to english language | 4373 |
| 42 | conference abstract.pt. | 5201624 |
| 43 | 41 not 42 | 1789 |
| 44 | limit 43 to yr="2014 -Current" | 1530 |
| 45 | remove duplicates from 44 | 1507 |

## Table S2. Electronic search strings for Ovid MEDLINE and Epub Ahead of Print, In-Process, In-Data-Review and Other Non-Indexed Citations, Daily and Versions

| # | Searches | Results |
| --- | --- | --- |
| 1 | exp nonalcoholic steatohepatitis/or (non-alcoholic steatohepatitis or nonalcoholic steatohepatitis or Metabolic dysfunction-associated steatohepatitis or NASH or MASH).mp. | 39293 |
| 2 | (F4 or cirrhosis).mp. | 166986 |
| 3 | ((Improvement adj5 fibrosis) or (cirrhosis adj5 regression) or alanine transaminase or ALT or aspartate transaminase or AST or decompens* or transplan* or hepatocellular carcinoma or HCC or ascites or haemorrhage or hemorrhage or encephalopathy or mortality or death).mp. | 3523088 |
| 4 | (NASH-CHECK or Short Form-36 or SF-36 or Short Form-6D or SF-6D or EQ-5D-5L or EQ-5D-5L-3L or EQ-5D or Chronic Liver Disease Questionnaire or CLDQ or Chronic Liver Disease Questionnaire-NASH or CLDQ-NASH or "Work Productivity and Activity Impairment" or WPAI or WPAI:SHP or activities of daily living or ADL or fatigue or pain or functional impairment or activity limitations or PROMIS-Fatigue).mp. | 1168477 |
| 5 | "health care cost"/or "drug cost"/or "hospital cost"/or "hospitalization cost"/or "nursing cost"/or ((health or global) adj2 burden).mp. or ((direct or indirect or societ* or employe*) adj2 (resource* or benefit*)).mp. or exp caregiver burden/or exp caregiver support/or (caregiver* or carer*).mp. or economics/or budget*.mp. or cost*.mp. or productivity/or productivity.mp. or absenteeism.mp. or absenteeism/or presenteeism.mp. or presenteeism/or "length of stay"/or Cost control/or (fiscal or financ* or funding).mp. or financial management.mp. or financial management/or health care utilization/or health care utili*.mp. or health care financing.mp. or health care financing/or health economics.mp. or health economics/or (burden adj2 (illness or disease$ or treatment*)).mp. or resource allocation/or budget/or pharmacoeconomics/or pharmacoeconomic*.mp. or pay?r.mp. or health care planning.mp. or health care planning/or (resource adj2 (use* or utili?ation or allocat* or burden or health)).mp. or (economic adj5 (burden or impact)).mp. or cost of illness.mp. or "cost of illness"/or cost control.mp. or "cost control"/or Economics, Medical/ | 1668824 |
| 6 | ("enhanced liver fibrosis" or ELF or FibroScan or FAST or "vibration-controlled transient elastography" or VCTE or "Fibrosis-4" or FIB-4 or "model for end-stage liver disease" or MELD or "NAFLD Activity Score" or NAS or non-invasive test*).mp. | 389040 |
| 7 | or/3-6 | 6281745 |
| 8 | Randomized Controlled Trials as Topic/or randomized controlled trial/or Random Allocation/or Double Blind Method/or Single Blind Method/or clinical trial/or (phase i* or phase 1*).mp. or (phase ii* or phase 2*).mp. or (phase iii* or phase 3*).mp. or (phase iv* or phase 4*).mp. or controlled clinical trial.mp. or randomized controlled trial.mp. or multicenter study.mp. or clinical trial.mp. or exp Clinical Trials as topic/or (clinical adj trial$).tw. or ((singl$ or doubl$ or treb$ or tripl$) adj (blind$3 or mask$3)).tw. or PLACEBOS/or placebo$.tw. or randomly allocated.tw. or (allocated adj2 random$).tw. or (single arm or single-arm or noncomparative or non-comparative).tw. | 2211913 |
| 9 | (Nonrandom* or non random* or non-random* or quasi-random* or quasirandom* or (phase adj3 (study or studies or trial*)) or ((crossover or cross-over) adj3 (study or studies or trial*)) or ((multicent* or multi-cent*) adj3 (study or studies or trial*))).ti,ab,hw,kf. or allocated.ti,ab,hw. or ((open label or open-label) adj5 (study or studies or trial*)).ti,ab,hw,kf. or ((equivalence or superiority or non-inferiority or noninferiority) adj3 (study or studies or trial*)).ti,ab,hw,kf. or (pragmatic study or pragmatic studies).ti,ab,hw,kf. or ((pragmatic or practical) adj3 trial*).ti,ab,hw,kf. or ((quasiexperimental or quasi-experimental) adj3 (study or studies or trial*)).ti,ab,hw,kf. or trial.ti,kf. | 1040518 |
| 10 | Epidemiologic studies/or exp case control studies/or exp cohort studies/or Case control.tw. or (cohort adj (study or studies)).tw. or Cohort analy$.tw. or (Follow up adj (study or studies)).tw. or (observational adj (study or studies)).tw. or Longitudinal.tw. or Retrospective.tw. or Cross sectional.tw. or Cross-sectional studies/or (registry or register$ or survey).ti,ab. or (real world or RWE).ti,ab. or Real-life.ti,ab. or exp seroepidemiologic studies/or (descriptive adj3 (study or studies or design or analysis or analyses)).ti,ab,kf. or ((multidimensional or (multi adj dimensional)) adj3 (study or studies or design or analysis or analyses)).ti,ab,kf. | 4940970 |
| 11 | or/8-10 | 6587608 |
| 12 | Case study/ | 2419664 |
| 13 | Case report.tw. | 432681 |
| 14 | Letter/ | 1263817 |
| 15 | or/12-14 | 3514766 |
| 16 | 11 not 15 | 6332065 |
| 17 | 1 and 2 and 7 and 16 | 2393 |
| 18 | (animal$ not human$).sh,hw. | 5197022 |
| 19 | 17 not 18 | 2366 |
| 20 | limit 19 to english language | 2323 |
| 21 | congress.pt. | 67729 |
| 22 | 20 not 21 | 2320 |
| 23 | limit 22 to yr="2014 -Current" | 2041 |
| 24 | remove duplicates from 23 | 2038 |

## Table S3. Electronic search strings for Cochrane (Cochrane Database of Systematic Review [CDSR], Database of Abstracts of Reviews of Effects [DARE], Cochrane Central Register of Controlled trials [CENTRAL], Cochrane Methodology Register [CMR], National Health Service Economic Evaluation Database [NHS EED], Health Technology Assessment [HTA] Database and the American College of Physicians [ACP] journal club)

| # | Searches | Results |
| --- | --- | --- |
| 1 | exp nonalcoholic steatohepatitis/or (non-alcoholic steatohepatitis or nonalcoholic steatohepatitis or Metabolic dysfunction-associated steatohepatitis or NASH or MASH).mp. | 3646 |
| 2 | (F4 or cirrhosis).mp. | 13478 |
| 3 | ((Improvement adj5 fibrosis) or (cirrhosis adj5 regression) or alanine transaminase or ALT or aspartate transaminase or AST or decompens* or transplan* or hepatocellular carcinoma or HCC or ascites or haemorrhage or hemorrhage or encephalopathy or mortality or death).mp. | 281925 |
| 4 | (NASH-CHECK or Short Form-36 or SF-36 or Short Form-6D or SF-6D or EQ-5D-5L or EQ-5D-5L-3L or EQ-5D or Chronic Liver Disease Questionnaire or CLDQ or Chronic Liver Disease Questionnaire-NASH or CLDQ-NASH or "Work Productivity and Activity Impairment" or WPAI or WPAI:SHP or activities of daily living or ADL or fatigue or pain or functional impairment or activity limitations or PROMIS-Fatigue).mp. | 330307 |
| 5 | "health care cost"/or "drug cost"/or "hospital cost"/or "hospitalization cost"/or "nursing cost"/or ((health or global) adj2 burden).mp. or ((direct or indirect or societ* or employe*) adj2 (resource* or benefit*)).mp. or exp caregiver burden/or exp caregiver support/or (caregiver* or carer*).mp. or economics/or budget*.mp. or cost*.mp. or productivity/or productivity.mp. or absenteeism.mp. or absenteeism/or presenteeism.mp. or presenteeism/or "length of stay"/or Cost control/or (fiscal or financ* or funding).mp. or financial management.mp. or financial management/or health care utilization/or health care utili*.mp. or health care financing.mp. or health care financing/or health economics.mp. or health economics/or (burden adj2 (illness or disease$ or treatment*)).mp. or resource allocation/or budget/or pharmacoeconomics/or pharmacoeconomic*.mp. or pay?r.mp. or health care planning.mp. or health care planning/or (resource adj2 (use* or utili?ation or allocat* or burden or health)).mp. or (economic adj5 (burden or impact)).mp. or cost of illness.mp. or "cost of illness"/or cost control.mp. or "cost control"/or Economics, Medical/ | 186149 |
| 6 | ("enhanced liver fibrosis" or ELF or FibroScan or FAST or "vibration-controlled transient elastography" or VCTE or "Fibrosis-4" or FIB-4 or "model for end-stage liver disease" or MELD or "NAFLD Activity Score" or NAS or non-invasive test*).mp. | 22636 |
| 7 | or/3-6 | 715044 |
| 8 | Randomized Controlled Trials as Topic/or randomized controlled trial/or Random Allocation/or Double Blind Method/or Single Blind Method/or clinical trial/or (phase i* or phase 1*).mp. or (phase ii* or phase 2*).mp. or (phase iii* or phase 3*).mp. or (phase iv* or phase 4*).mp. or controlled clinical trial.mp. or randomized controlled trial.mp. or multicenter study.mp. or clinical trial.mp. or exp Clinical Trials as topic/or (clinical adj trial$).tw. or ((singl$ or doubl$ or treb$ or tripl$) adj (blind$3 or mask$3)).tw. or PLACEBOS/or placebo$.tw. or randomly allocated.tw. or (allocated adj2 random$).tw. or (single arm or single-arm or noncomparative or non-comparative).tw. | 1275651 |
| 9 | (Nonrandom* or non random* or non-random* or quasi-random* or quasirandom* or (phase adj3 (study or studies or trial*)) or ((crossover or cross-over) adj3 (study or studies or trial*)) or ((multicent* or multi-cent*) adj3 (study or studies or trial*))).ti,ab,hw,kf. or allocated.ti,ab,hw. or ((open label or open-label) adj5 (study or studies or trial*)).ti,ab,hw,kf. or ((equivalence or superiority or non-inferiority or noninferiority) adj3 (study or studies or trial*)).ti,ab,hw,kf. or (pragmatic study or pragmatic studies).ti,ab,hw,kf. or ((pragmatic or practical) adj3 trial*).ti,ab,hw,kf. or ((quasiexperimental or quasi-experimental) adj3 (study or studies or trial*)).ti,ab,hw,kf. or trial.ti,kf. | 794230 |
| 10 | Epidemiologic studies/or exp case control studies/or exp cohort studies/or Case control.tw. or (cohort adj (study or studies)).tw. or Cohort analy$.tw. or (Follow up adj (study or studies)).tw. or (observational adj (study or studies)).tw. or Longitudinal.tw. or Retrospective.tw. or Cross sectional.tw. or Cross-sectional studies/or (registry or register$ or survey).ti,ab. or (real world or RWE).ti,ab. or Real-life.ti,ab. or exp seroepidemiologic studies/or (descriptive adj3 (study or studies or design or analysis or analyses)).ti,ab,kf. or ((multidimensional or (multi adj dimensional)) adj3 (study or studies or design or analysis or analyses)).ti,ab,kf. | 393590 |
| 11 | or/8-10 | 1510886 |
| 12 | Case study/ | 0 |
| 13 | Case report.tw. | 3167 |
| 14 | Letter/ | 0 |
| 15 | or/12-14 | 3167 |
| 16 | 11 not 15 | 1508442 |
| 17 | 1 and 2 and 7 and 16 | 542 |
| 18 | (animal$ not human$).sh,hw. | 3370 |
| 19 | 17 not 18 | 542 |
| 20 | limit 19 to english language | 541 |
| 21 | conference abstract.pt. | 0 |
| 22 | 20 not 21 | 541 |
| 23 | limit 22 to yr="2014 -Current" | 489 |
| 24 | remove duplicates from 23 | 466 |

## Table S4. List of eligible studies after full-text review (*n* = 317) grouped by inclusion and exclusion from data extraction

| **First author (last name)** | **Year** | **Title** | **Reason for excluding from data extraction (if applicable)** |
| --- | --- | --- | --- |
| **Studies included for data extraction (*n* = 95)** | | | |
| Alam | 2023 | Estimating the cost of illness of non-alcoholic fatty liver disease in Bangladesh | **–** |
| Alkhouri | 2016 | Liver transplantation for nonalcoholic steatohepatitis in young patients | **–** |
| Andrade | 2022 | Risk predictors of advanced hepatic fibrosis in patients with nonalcoholic fatty liver disease - a survey in a university hospital in Brazil | **–** |
| Are | 2021 | Enhanced Liver Fibrosis Score Can Be Used to Predict Liver-Related Events in Patients With Nonalcoholic Steatohepatitis and Compensated Cirrhosis | **–** |
| Axley | 2019 | NASH Is the Most Rapidly Growing Etiology for Acute-on-Chronic Liver Failure-Related Hospitalization and Disease Burden in the United States: A Population-Based Study | **–** |
| Becker | 2024 | Disparities in Liver Transplantation for Nonalcoholic Steatohepatitis in Women | **–** |
| Bertot | 2018 | Diabetes impacts prediction of cirrhosis and prognosis by non-invasive fibrosis models in non-alcoholic fatty liver disease | **–** |
| Boursier | 2020 | Hospitalization costs and risk of mortality in adults with nonalcoholic steatohepatitis: Analysis of a French national hospital database | **–** |
| Brandman | 2022 | Comparison of clinical prediction rules for ruling out cirrhosis in nonalcoholic fatty liver disease (NAFLD) | **–** |
| Bril | 2024 | Noninvasive tests to identify liver fibrosis in metabolic dysfunction-associated steatotic liver disease are affected by race | **–** |
| Calzadilla-Bertot | 2021 | ABIDE: An Accurate Predictive Model of Liver Decompensation in Patients With Nonalcoholic Fatty Liver-Related Cirrhosis | **–** |
| Canbay | 2021 | Healthcare resource utilization and costs among nonalcoholic fatty liver disease patients in Germany | **–** |
| Chang | 2023 | Machine learning models are superior to noninvasive tests in identifying clinically significant stages of NAFLD and NAFLD-related cirrhosis | **–** |
| Cholankeril | 2023 | Longitudinal changes in fibrosis markers are associated with risk of cirrhosis and hepatocellular carcinoma in non-alcoholic fatty liver disease | **–** |
| Chu | 2024 | Underrecognition and Suboptimal Quality of Care for Nonalcoholic Fatty Liver Disease Cirrhosis in Primary Care Patients with Diabetes Mellitus | **–** |
| Doycheva | 2018 | Nonalcoholic Steatohepatitis is the Most Rapidly Increasing Indication for Liver Transplantation in Young Adults in the United States | **–** |
| Eddowes | 2019 | Accuracy of FibroScan Controlled Attenuation Parameter and Liver Stiffness Measurement in Assessing Steatosis and Fibrosis in Patients With Nonalcoholic Fatty Liver Disease | **–** |
| Eshraghian | 2022 | Nonalcoholic Steatohepatitis Is the Most Rapidly Growing Indication for Liver Transplantation in Iranian Patients | **–** |
| Ferrarese | 2022 | Nash Up, Virus Down: How the Waiting List Is Changing for Liver Transplantation: A Single Center Experience from Italy | **–** |
| Fishman | 2024 | Cost burden of cirrhosis and liver disease progression in metabolic dysfunction-associated steatohepatitis: A US cohort study | **–-** |
| Frenette | 2021 | Emricasan to prevent new decompensation in patients with NASH-related decompensated cirrhosis | **–** |
| Garcia-Tsao | 2020 | Randomized placebo-controlled trial of emricasan for non-alcoholic steatohepatitis-related cirrhosis with severe portal hypertension | **–** |
| Gerhardt | 2020 | Biopsy rate and nonalcoholic steatohepatitis (NASH) in patients with nonalcoholic fatty liver disease (NAFLD) | **–** |
| Gordon | 2020 | Disease Severity Is Associated With Higher Healthcare Utilization in Nonalcoholic Steatohepatitis Medicare Patients | **–** |
| Gordon | 2020 | Health Care Use and Costs Among Patients With Nonalcoholic Steatohepatitis With Advanced Fibrosis Using the Fibrosis-4 Score | **–** |
| Govaere | 2020 | Transcriptomic profiling across the nonalcoholic fatty liver disease spectrum reveals gene signatures for steatohepatitis and fibrosis | **–** |
| Hagstrom | 2020 | Ability of Noninvasive Scoring Systems to Identify Individuals in the Population at Risk for Severe Liver Disease | **–** |
| Harrison | 2018 | Simtuzumab Is Ineffective for Patients With Bridging Fibrosis or Compensated Cirrhosis Caused by Nonalcoholic Steatohepatitis | **–** |
| Harrison | 2020 | Selonsertib for patients with bridging fibrosis or compensated cirrhosis due to NASH: Results from randomized phase III STELLAR trials | **–** |
| Hirode | 2019 | Increasing Clinical and Economic Burden of Nonalcoholic Fatty Liver Disease Among Hospitalized Adults in the United States | **–** |
| Holdsworth | 2023 | Impact of neighbourhood-level social determinants of health on healthcare utilisation and perinatal outcomes in pregnant women with NAFLD cirrhosis: a population-based study in Ontario, Canada | **–** |
| Holmer | 2018 | Nonalcoholic fatty liver disease is an increasing indication for liver transplantation in the Nordic countries | **–** |
| Howell | 2016 | The increasing burden of potentially preventable liver disease among adult liver transplant recipients: A comparative analysis of liver transplant indication by era in Australia and New Zealand | **–** |
| Imajo | 2022 | Direct Comparison of US and MR Elastography for Staging Liver Fibrosis in Patients With Nonalcoholic Fatty Liver Disease | **–** |
| Jaafar | 2019 | Fibroscan and low-density lipoprotein as determinants of severe liver fibrosis in diabetic patients with nonalcoholic fatty liver disease | **–** |
| Kaplan | 2024 | Clinical and genetic risk factors for progressive fibrosis in metabolic dysfunction-associated steatotic liver disease | **–** |
| Karlas | 2018 | Impact of controlled attenuation parameter on detecting fibrosis using liver stiffness measurement | **–** |
| Karnam | 2023 | Impact of living donor liver transplantation on long-term cardiometabolic and graft outcomes in cirrhosis due to nonalcoholic steatohepatitis | **–** |
| Kern | 2019 | High incidence of hepatocellular carcinoma and postoperative complications in patients with nonalcoholic steatohepatitis as a primary indication for deceased liver transplantation | **–** |
| Khajehahmadi | 2024 | Increasing Prevalence and High Survival Rate of Liver Transplanted Patients with NASH and PSC Cirrhosis | **–** |
| Kim | 2019 | Trends in hospitalizations for chronic liver disease-related liver failure in the United States, 2005-2014 | **–** |
| Koch | 2024 | H2FPEF Scores Are Increased in Patients with NASH Cirrhosis and Are Associated with Post-liver Transplant Heart Failure | **–** |
| Lee | 2024 | Etiology-Specific Effects of Impaired Functional Status on Liver Transplant Outcomes | **–** |
| Liu | 2021 | A prospective 5-year study on the use of transient elastography to monitor the improvement of non-alcoholic fatty liver disease following bariatric surgery | **–** |
| Loomba | 2023 | Semaglutide 2.4 mg once weekly in patients with non-alcoholic steatohepatitis-related cirrhosis: a randomised, placebo-controlled phase 2 trial | **–** |
| Loomba | 2023 | Liver stiffness thresholds to predict disease progression and clinical outcomes in bridging fibrosis and cirrhosis | **–** |
| Mathews | 2022 | Prevalence and Financial Burden of Digestive Diseases in a Commercially Insured Population | **–** |
| Matsumoto | 2021 | Ultrasonographic grayscale findings related to fibrosis in patients with non-alcoholic fatty liver disease: comparison with transient elastography and Fib-4 index | **–** |
| Mozes | 2023 | Performance of non-invasive tests and histology for the prediction of clinical outcomes in patients with non-alcoholic fatty liver disease: an individual participant data meta-analysis | **–** |
| Mozes | 2024 | Diagnostic accuracy of non-invasive tests to screen for at-risk MASH-An individual participant data meta-analysis | **–** |
| Nagai | 2019 | Increased Risk of Death in First Year After Liver Transplantation Among Patients With Nonalcoholic Steatohepatitis vs Liver Disease of Other Etiologies | **–** |
| Noureddin | 2023 | Increased accuracy in identifying NAFLD with advanced fibrosis and cirrhosis: independent validation of the Agile 3+ and 4 scores | **–** |
| Oladunjoye | 2021 | A Retrospective Study of Hospitalizations in the USA: Proportion of Hospitalizations With Non-Alcoholic Fatty Liver Disease in Non-Obese Population | **–** |
| Paik | 2023 | Non-alcoholic fatty liver disease is associated with greater risk of 30-day hospital readmission in the United States (U.S.) | **–** |
| Parrish | 2019 | The Changing Face of Liver Transplantation in the United States: The Effect of HCV Antiviral Eras on Transplantation Trends and Outcomes | **–** |
| Petta | 2020 | Healthcare resource utilization and costs of nonalcoholic steatohepatitis patients with advanced liver disease in Italy | **–** |
| Pinyopornpanish | 2021 | Chemopreventive Effect of Statin on Hepatocellular Carcinoma in Patients With Nonalcoholic Steatohepatitis Cirrhosis | **–** |
| Podrug | 2021 | Diagnostic Performance of 2-D Shear-Wave Elastography with Propagation Maps and Attenuation Imaging in Patients with Non-Alcoholic Fatty Liver Disease | **–** |
| Qayed | 2023 | Characteristics and Outcomes of Black and White Patients Hospitalized With Nonalcoholic Steatohepatitis: A Nationwide Analysis | **–** |
| Qazi-Arisar | 2022 | Divergent trajectories of lean vs obese non-alcoholic steatohepatitis patients from listing to post-transplant: A retrospective cohort study | **–** |
| Quillin | 2014 | Increasing prevalence of nonalcoholic steatohepatitis as an indication for liver transplantation | **–** |
| Reznicek | 2023 | Outcomes of Liver Transplantation in Patients With Preexisting Coronary Artery Disease | **–** |
| Rinella | 2023 | Factors Impacting Survival in Those Transplanted for NASH Cirrhosis: Data From the NailNASH Consortium | **–** |
| Rinella | 2024 | A randomized, double-blind, placebo-controlled trial of aldafermin in patients with NASH and compensated cirrhosis | **–** |
| Romero-Gomez | 2020 | Disease severity predicts higher healthcare costs among hospitalized nonalcoholic fatty liver disease/nonalcoholic steatohepatitis (NAFLD/NASH) patients in Spain | **–** |
| Rudolfsen | 2024 | Societal costs and survival of patients with biopsy-verified non-alcoholic steatohepatitis: Danish nationwide register-based study | **–** |
| Salman | 2021 | Impact of laparoscopic sleeve gastrectomy on fibrosis stage in patients with child-A NASH-related cirrhosis | **–** |
| Sanyal | 2022 | Cirrhosis regression is associated with improved clinical outcomes in patients with nonalcoholic steatohepatitis | **–** |
| Sanyal | 2023 | Diagnostic performance of circulating biomarkers for non-alcoholic steatohepatitis | **–** |
| Seko | 2023 | Diagnostic accuracy of enhanced liver fibrosis test for nonalcoholic steatohepatitis-related fibrosis: Multicenter study | **–** |
| Shabbir | 2024 | Role of Alanine Transaminase and Transient Elastography in Categorising Nonalcoholic Fatty Liver Disease Subgroups | **–** |
| Shingina | 2019 | Future Trends in Demand for Liver Transplant: Birth Cohort Effects Among Patients With NASH and HCC | **–** |
| Srivastava | 2019 | Prospective evaluation of a primary care referral pathway for patients with non-alcoholic fatty liver disease | **–** |
| Tan | 2021 | Poor Outcomes of Cirrhosis due to Nonalcoholic Steatohepatitis Compared With Hepatitis B After Decompensation With Ascites | **–** |
| Tang | 2023 | N-terminal propeptide of type 3 collagen-based sequential algorithm can identify high-risk steatohepatitis and fibrosis in MAFLD | **–** |
| Taru | 2024 | FAST and Agile-the MASLD drift: Validation of Agile 3+, Agile 4 and FAST scores in 246 biopsy-proven NAFLD patients meeting MASLD criteria of prevalent caucasian origin | **–** |
| Thuluvath | 2019 | Poor Survival After Retransplantation in NASH Cirrhosis | **–** |
| Thuluvath | 2019 | Waiting List Mortality and Transplant Rates for NASH Cirrhosis When Compared With Cryptogenic, Alcoholic, or AIH Cirrhosis | **–** |
| Tuong | 2020 | Non-Alcoholic Fatty Liver Disease in Patients with Type 2 Diabetes: Evaluation of Hepatic Fibrosis and Steatosis Using Fibroscan | **–** |
| Vieira Barbosa | 2022 | Fibrosis-4 Index as an Independent Predictor of Mortality and Liver-Related Outcomes in NAFLD | **–** |
| Vijayaraghavan | 2023 | Intragastric Balloon in Obese Compensated Nonalcoholic Steatohepatitis Cirrhosis Patients Is Safe and Achieves Significant Weight Reduction at 6-Months | **–** |
| Vilar-Gomez | 2021 | Type 2 Diabetes and Metformin Use Associate With Outcomes of Patients With Nonalcoholic Steatohepatitis-Related, Child-Pugh A Cirrhosis | **–** |
| Villeret | 2022 | Liver transplantation for NAFLD cirrhosis: Age and recent coronary angioplasty are major determinants of survival | **–** |
| Villeret | 2023 | Inevitability of disease recurrence after liver transplantation for NAFLD cirrhosis | **–** |
| Wang | 2022 | Serum Fibrosis Tests Guide Prognosis in Metabolic Dysfunction-Associated Fatty Liver Disease Patients Referred From Primary Care | **–** |
| Wong | 2019 | Unified interpretation of liver stiffness measurement by M and XL probes in non-alcoholic fatty liver disease | **–** |
| Wong | 2021 | Real-world Comorbidity Burden, Health Care Utilization, and Costs of Nonalcoholic Steatohepatitis Patients With Advanced Liver Diseases | **–** |
| Younossi | 2016 | The economic and clinical burden of nonalcoholic fatty liver disease in the United States and Europe | **–** |
| Younossi | 2019 | Reduced Patient-Reported Outcome Scores Associate With Level of Fibrosis in Patients With Nonalcoholic Steatohepatitis | **–** |
| Younossi | 2019 | Validation of Chronic Liver Disease Questionnaire for Nonalcoholic Steatohepatitis in Patients With Biopsy-Proven Nonalcoholic Steatohepatitis | **–** |
| Younossi | 2020 | Fatigue and Pruritus in Patients with Advanced Fibrosis Due to Nonalcoholic Steatohepatitis: The Impact on Patient-Reported Outcomes | **–** |
| Younossi | 2021 | The Association of Histologic and Noninvasive Tests With Adverse Clinical and Patient-Reported Outcomes in Patients With Advanced Fibrosis Due to Nonalcoholic Steatohepatitis | **–** |
| Younossi | 2021 | Improvements of Fibrosis and Disease Activity Are Associated With Improvement of Patient-Reported Outcomes in Patients With Advanced Fibrosis Due to Nonalcoholic Steatohepatitis | **–** |
| Younossi | 2021 | Nonalcoholic Steatohepatitis Is the Most Rapidly Increasing Indication for Liver Transplantation in the United States | **–** |
| Zou | 2020 | A Nationwide Study of Inpatient Admissions, Mortality, and Costs for Patients with Cirrhosis from 2005 to 2015 in the USA | **–** |
| **Studies excluded from data extraction (n = 222)** | | |  |
| Abdelmalek | 2024 | Pegbelfermin in Patients With Nonalcoholic Steatohepatitis and Compensated Cirrhosis (FALCON 2): A Randomized Phase 2b Study | Only reports NITs not listed in PICOS |
| Abeysekera | 2020 | Prevalence of steatosis and fibrosis in young adults in the UK: a population-based study | 20 or fewer patients |
| Ahmed | 2020 | Predictive Factors and Time to Development of Hepatic Decompensation in Patients with Non-alcoholic Fatty Liver Disease | Only reports epidemiology outcomes |
| Ajmera | 2022 | Liver Stiffness on Magnetic Resonance Elastography and the MEFIB Index and Liver-Related Outcomes in Nonalcoholic Fatty Liver Disease: A Systematic Review and Meta-Analysis of Individual Participants | SLR |
| Ajmera | 2023 | Longitudinal association between overweight years, polygenic risk and NAFLD, significant fibrosis and cirrhosis | Only reports epidemiology outcomes |
| Ajmera | 2023 | A prospective study on the prevalence of NAFLD, advanced fibrosis, cirrhosis and hepatocellular carcinoma in people with type 2 diabetes | Only reports epidemiology outcomes |
| Akuta | 2016 | Relationships between Genetic Variations of PNPLA3, TM6SF2 and Histological Features of Nonalcoholic Fatty Liver Disease in Japan | 20 or fewer patients |
| Albhaisi | 2023 | Fibrosis-4 index is associated with the risk of hepatocellular carcinoma in patients with cirrhosis and nonalcoholic steatohepatitis | Only reports epidemiology outcomes |
| Alexander | 2019 | Risks and clinical predictors of cirrhosis and hepatocellular carcinoma diagnoses in adults with diagnosed NAFLD: Real-world study of 18 million patients in four European cohorts | Only reports epidemiology outcomes |
| Alkhouri | 2018 | Serum Wisteria floribunda agglutinin-positive Mac-2-binding protein levels predict the presence of fibrotic nonalcoholic steatohepatitis (NASH) and NASH cirrhosis | Only reports epidemiology outcomes |
| Allen | 2022 | Clinical course of non-alcoholic fatty liver disease and the implications for clinical trial design | Only reports epidemiology outcomes |
| Almomani | 2023 | Epidemiology and prevalence of lean nonalcoholic fatty liver disease and associated cirrhosis, hepatocellular carcinoma, and cardiovascular outcomes in the United States: a population-based study and review of literature | Only reports epidemiology outcomes |
| Amernia | 2021 | FIB-4, APRI, and AST/ALT ratio compared to FibroScan for the assessment of hepatic fibrosis in patients with non-alcoholic fatty liver disease in Bandar Abbas, Iran | 20 or fewer patients |
| Amoroso | 2024 | Non-invasive biomarkers prognostic of decompensation events in NASH cirrhosis: a systematic literature review | SLR |
| Ampuero | 2021 | Definite and indeterminate nonalcoholic steatohepatitis share similar clinical features and prognosis: A longitudinal study of 1893 biopsy-proven nonalcoholic fatty liver disease subjects | Only reports epidemiology outcomes |
| Ampuero | 2024 | The biochemical pattern defines MASLD phenotypes linked to distinct histology and prognosis | Only reports epidemiology outcomes |
| Angulo | 2015 | Liver Fibrosis, but No Other Histologic Features, Is Associated With Long-term Outcomes of Patients With Nonalcoholic Fatty Liver Disease | Only reports epidemiology outcomes |
| Anushiravani | 2023 | Multicenter validation of FIB-6 as a novel machine learning non-invasive score to rule out liver cirrhosis in biopsy-proven MAFLD | Only reports NITs not listed in PICOS |
| Arasaradnam | 2015 | Non-invasive distinction of non-alcoholic fatty liver disease using urinary volatile organic compound analysis: Early results | Only reports NITs not listed in PICOS |
| Barritt | 2021 | Patient Determinants for Histologic Diagnosis of NAFLD in the Real World: A TARGET-NASH Study | Only reports epidemiology outcomes |
| Bassegoda | 2022 | High frequency of acute decompensation and cancer in patients with compensated cirrhosis due to nonalcoholic fatty liver disease: A retrospective cohort study | Only reports epidemiology outcomes |
| Bauer | 2023 | Point Shear Wave Elastography by ElastPQ for Fibrosis Screening in Patients with NAFLD: A Prospective, Multicenter Comparison to Vibration-Controlled Elastography | 20 or fewer patients |
| Beste | 2015 | Trends in Burden of Cirrhosis and Hepatocellular Carcinoma by Underlying Liver Disease in US Veterans, 2001-2013 | Only reports epidemiology outcomes |
| Bhadoria | 2017 | Impact of family history of metabolic traits on severity of non-alcoholic steatohepatitis related cirrhosis: A cross-sectional study | Only reports epidemiology outcomes |
| Bridi | 2024 | The impact of genetic risk on the prevalence of advanced fibrosis and cirrhosis in prospectively assessed patients with type 2 diabetes | 20 or fewer patients |
| Buzzetti | 2019 | Collagen proportionate area is an independent predictor of long-term outcome in patients with non-alcoholic fatty liver disease | Only reports NITs not listed in PICOS |
| Canbay | 2020 | Patterns and predictors of mortality and disease progression among patients with non-alcoholic fatty liver disease | Only reports epidemiology outcomes |
| Cao | 2022 | Accuracy of controlled attenuation parameter (CAP) and liver stiffness measurement (LSM) for assessing steatosis and fibrosis in non-alcoholic fatty liver disease: A systematic review and meta-analysis | SLR |
| Cao | 2023 | Sex- and reproductive status-specific relationships between body composition and non-alcoholic fatty liver disease | Only reports epidemiology outcomes |
| Carias | 2016 | Nonalcoholic steatohepatitis is strongly associated with sarcopenic obesity in patients with cirrhosis undergoing liver transplant evaluation | Only reports epidemiology outcomes |
| Castello | 2019 | Post-transplantation outcome in non-alcoholic steatohepatitis cirrhosis: Comparison with alcoholic cirrhosis | 20 or fewer patients |
| Chan | 2014 | Progression of liver disease in non-alcoholic fatty liver disease: A prospective clinicopathological follow-up study | 20 or fewer patients |
| Chan | 2020 | Positive Hepatitis B Core Antibody Is Associated With Cirrhosis and Hepatocellular Carcinoma in Nonalcoholic Fatty Liver Disease | Only reports epidemiology outcomes |
| Chhatwal | 2022 | Analysis of a Simulation Model to Estimate Long-term Outcomes in Patients with Nonalcoholic Fatty Liver Disease | Only reports epidemiology outcomes |
| Conway | 2023 | Integration of deep learning-based histopathology and transcriptomics reveals key genes associated with fibrogenesis in patients with advanced NASH | Only reports epidemiology outcomes |
| Corey | 2017 | Risk factors for hepatocellular carcinoma in cirrhosis due to nonalcoholic fatty liver disease: A multicenter, case-control study | Only reports epidemiology outcomes |
| Cortes | 2022 | Incidentalomas are associated with an increase in liver transplantation in patients with cirrhosis: a single-center retrospective study | Only reports epidemiology outcomes |
| Cotrim | 2016 | Nonalcoholic steatohepatitis and hepatocellular carcinoma: Brazilian survey | Only reports epidemiology outcomes |
| Cui | 2015 | Comparative diagnostic accuracy of magnetic resonance elastography vs. eight clinical prediction rules for non-invasive diagnosis of advanced fibrosis in biopsy-proven non-alcoholic fatty liver disease: A prospective study | 20 or fewer patients |
| Danford | 2019 | Evidence of bias during liver transplant evaluation of non-alcoholic steatohepatitis cirrhosis patients | Only reports epidemiology outcomes |
| Daniels | 2019 | ADAPT: An Algorithm Incorporating PRO-C3 Accurately Identifies Patients With NAFLD and Advanced Fibrosis | Only reports NITs not listed in PICOS |
| De Roza | 2021 | Immunoglobulin G in non-alcoholic steatohepatitis predicts clinical outcome: A prospective multi-centre cohort study | Only reports epidemiology outcomes |
| Deng | 2024 | Associations of non-alcoholic fatty liver disease and cirrhosis with liver cancer in European and East Asian populations: A Mendelian randomization study | Only reports epidemiology outcomes |
| Du Plessis | 2016 | Pro-inflammatory cytokines but not endotoxin-related parameters associate with disease severity in patients with NAFLD | 20 or fewer patients |
| Duseja | 2022 | Clinicopathological Profile and Outcome of a Large Cohort of Patients with Nonalcoholic Fatty Liver Disease from South Asia: Interim Results of the Indian Consortium on Nonalcoholic Fatty Liver Disease | Only reports epidemiology outcomes |
| Elias | 2018 | Serum immunoglobulin a levels and non-alcoholic fatty liver disease | Only reports epidemiology outcomes |
| El-Serag | 2023 | Bioimpedance analysis predicts the etiology of cirrhosis in a prospective cohort study | Only reports epidemiology outcomes |
| Enooku | 2020 | Hepatic FATP5 expression is associated with histological progression and loss of hepatic fat in NAFLD patients | Only reports epidemiology outcomes |
| Esquivel | 2018 | Laparoscopic Sleeve Gastrectomy Resolves NAFLD: Another Formal Indication for Bariatric Surgery? | 20 or fewer patients |
| Estes | 2020 | Modelling NAFLD disease burden in four Asian regions-2019-2030 | Only reports epidemiology outcomes |
| Feldman | 2021 | Liver-related mortality is increased in lean subjects with nonalcoholic fatty liver disease compared to overweight and obese subjects | 20 or fewer patients |
| Ferri | 2020 | Reduced lysosomal acid lipase activity in blood and platelets is associated with nonalcoholic fatty liver disease | Only reports epidemiology outcomes |
| Flemming | 2021 | NAFLD and Alcohol-Associated Liver Disease Will Be Responsible for Almost All New Diagnoses of Cirrhosis in Canada by 2040 | Only reports epidemiology outcomes |
| Forlano | 2024 | A prospective study on the prevalence of MASLD in people with type-2 diabetes in the community. Cost effectiveness of screening strategies | 20 or fewer patients |
| Friden | 2024 | Substitutions of saturated fat intakes with other macronutrients and foods and risk of NAFLD cirrhosis and all-cause hepatocellular carcinoma: a prospective cohort study | Only reports epidemiology outcomes |
| Gadd | 2016 | Altered Peripheral Blood Monocyte Phenotype and Function in Chronic Liver Disease: Implications for Hepatic Recruitment and Systemic Inflammation | Only reports epidemiology outcomes |
| Gawrieh | 2019 | Histologic Findings of Advanced Fibrosis and Cirrhosis in Patients With Nonalcoholic Fatty Liver Disease Who Have Normal Aminotransferase Levels | Only reports NITs not listed in PICOS |
| Gawrieh | 2024 | Increases and decreases in liver stiffness measurement are independently associated with the risk of liver-related events in NAFLD | Only reports epidemiology outcomes |
| Geier | 2023 | Clinical characteristics of patients with non-alcoholic fatty liver disease (NAFLD) in Germany - First data from the German NAFLD-Registry | Only reports epidemiology outcomes |
| Gidener | 2021 | Liver Stiffness by Magnetic Resonance Elastography Predicts Future Cirrhosis, Decompensation, and Death in NAFLD | Only reports NITs not listed in PICOS |
| Gidener | 2023 | Change in serial liver stiffness measurement by magnetic resonance elastography and outcomes in NAFLD | Only reports NITs not listed in PICOS |
| Goldberg | 2017 | Changes in the Prevalence of Hepatitis C Virus Infection, Nonalcoholic Steatohepatitis, and Alcoholic Liver Disease Among Patients With Cirrhosis or Liver Failure on the Waitlist for Liver Transplantation | Only reports epidemiology outcomes |
| Gosnell | 2024 | Disparate outcomes in Hispanic patients with metabolic dysfunction associated steatotic liver disease/steatohepatitis and type 2 diabetes: Large cohort study | Only reports epidemiology outcomes |
| Ha | 2023 | Mortality and Liver-Related Events in Lean Versus Non-Lean Nonalcoholic Fatty Liver Disease: A Systematic Review and Meta-analysis | SLR |
| Haflidadottir | 2014 | Long-term follow-up and liver-related death rate in patients with non-alcoholic and alcoholic related fatty liver disease | Only reports epidemiology outcomes |
| Hagstrom | 2017 | Fibrosis stage but not NASH predicts mortality and time to development of severe liver disease in biopsy-proven NAFLD | 20 or fewer patients |
| Han | 2020 | MR elastography-based liver fibrosis correlates with liver events in nonalcoholic fatty liver patients: A multicenter study | Only reports NITs not listed in PICOS |
| Harrison | 2021 | Prospective evaluation of the prevalence of non-alcoholic fatty liver disease and steatohepatitis in a large middle-aged US cohort | Only reports epidemiology outcomes |
| Harrison | 2023 | A randomized, double-blind, placebo-controlled phase IIa trial of efruxifermin for patients with compensated NASH cirrhosis | Clinical study with 50 or fewer patients |
| Hoehn | 2015 | Effect of pretransplant diabetes on short-term outcomes after liver transplantation: A National cohort study | Only reports epidemiology outcomes |
| Holmer | 2022 | Effect of common genetic variants on the risk of cirrhosis in non-alcoholic fatty liver disease during 20 years of follow-up | Only reports epidemiology outcomes |
| Hussain | 2020 | Decompensated cirrhosis is the commonest presentation for NAFLD patients undergoing liver transplant assessment | Only reports epidemiology outcomes |
| Idilman | 2021 | Natural history of cirrhosis: Changing trends in etiology over the years | Only reports epidemiology outcomes |
| Ioannou | 2019 | Models estimating risk of hepatocellular carcinoma in patients with alcohol or NAFLD-related cirrhosis for risk stratification | Only reports epidemiology outcomes |
| Izzy | 2021 | Bariatric surgery proves long-term benefit in patients with cirrhosis | 20 or fewer patients |
| Jang | 2020 | Changes in Characteristics of Patients with Liver Cirrhosis Visiting a Tertiary Hospital over 15 Years: a Retrospective Multi-Center Study in Korea | Only reports epidemiology outcomes |
| Jarvis | 2020 | Metabolic risk factors and incident advanced liver disease in non-alcoholic fatty liver disease (NAFLD): A systematic review and meta-analysis of population-based observational studies | SLR |
| Jordens | 2021 | Pulmonary hypertension is associated with an increased incidence of NAFLD: A retrospective cohort study of 18,910 patients | Only reports epidemiology outcomes |
| Kabbany | 2017 | Prevalence of Nonalcoholic Steatohepatitis-Associated Cirrhosis in the United States: An Analysis of National Health and Nutrition Examination Survey Data | Only reports epidemiology outcomes |
| Kakar | 2019 | Incidence of Recurrent NASH-Related Allograft Cirrhosis | Only reports epidemiology outcomes |
| Kanwal | 2018 | Risk of Hepatocellular Cancer in Patients With Non-Alcoholic Fatty Liver Disease | Only reports epidemiology outcomes |
| Kanwal | 2020 | Effect of Metabolic Traits on the Risk of Cirrhosis and Hepatocellular Cancer in Nonalcoholic Fatty Liver Disease | Only reports epidemiology outcomes |
| Kanwal | 2023 | Risk factors for HCC in contemporary cohorts of patients with cirrhosis | Only reports epidemiology outcomes |
| Kao | 2020 | Fibroscan-Based Score to Predict Significant Liver Fibrosis in Morbidly Obese Patients with Nonalcoholic Fatty Liver Disease | 20 or fewer patients |
| Karnam | 2023 | Predicting the outcome of liver transplantation in patients with non-alcoholic steatohepatitis cirrhosis: The NASH LT risk-benefit calculator | Only reports NITs not listed in PICOS |
| Kawanaka | 2023 | Soluble CD163 is a predictor of fibrosis and hepatocellular carcinoma development in nonalcoholic steatohepatitis | Clinical study with 50 or fewer patients |
| Kaze | 2020 | The changing pattern of cirrhosis in Belgium: a study based on two cohorts prospectively collected 15 years apart | Only reports epidemiology outcomes |
| Kennedy-Martin | 2017 | Health-related quality of life burden of nonalcoholic steatohepatitis: a robust pragmatic literature review | SLR |
| Kessoku | 2014 | Simple scoring system for predicting cirrhosis in nonalcoholic fatty liver disease | Clinical study with 50 or fewer patients |
| Khajehahmadi | 2021 | Aspartate Aminotransferase-To-Platelet Ratio Index: a Potential Predictor of Prognosis in the Most Common Types of Cirrhosis | Clinical study with 50 or fewer patients |
| Khajehahmadi | 2023 | Non-Alcoholic Steatohepatitis and Autoimmune Hepatitis Cirrhosis May Have a Higher Risk of Progression to Hepatocellular Carcinoma | 20 or fewer patients |
| Kim | 2023 | Magnetic resonance elastography-based prediction model for hepatic decompensation in NAFLD: A multicenter cohort study | Only reports NITs not listed in PICOS |
| Kirchmeyer | 2023 | Altered profiles of circulating cytokines in chronic liver diseases (NAFLD/HCC): Impact of the PNPLA3I148M risk allele | Only reports epidemiology outcomes |
| Kitajima | 2020 | Liver transplant waitlist outcomes in alcoholic hepatitis compared with other liver diseases: An analysis of UNOS registry | Only reports epidemiology outcomes |
| Kleiner | 2019 | Association of Histologic Disease Activity With Progression of Nonalcoholic Fatty Liver Disease | Only reports epidemiology outcomes |
| Labenz | 2022 | Derivation and validation of the nonalcoholic fatty liver disease cirrhosis score (NCS) to distinguish bridging fibrosis from cirrhosis | Only reports NITs not listed in PICOS |
| Ladner | 2024 | Increasing prevalence of cirrhosis among insured adults in the United States, 2012-2018 | Only reports epidemiology outcomes |
| Lavrado | 2024 | Impact of PNPLA3 and TM6SF2 polymorphisms on the prognosis of patients with MASLD and type 2 diabetes mellitus | Only reports epidemiology outcomes |
| Le | 2024 | Global incidence of adverse clinical events in nonalcoholic fatty liver disease: A systematic review and meta-analysis | SLR |
| Lee | 2024 | Low liver fat in non-alcoholic steatohepatitis-related significant fibrosis and cirrhosis is associated with hepatocellular carcinoma, decompensation and mortality | SLR |
| Leung | 2015 | Characteristics of hepatocellular carcinoma in cirrhotic and non-cirrhotic non-alcoholic fatty liver disease | Only reports epidemiology outcomes |
| Lim | 2023 | Ethnic disparities in waitlist outcomes of patients with nonalcoholic steatohepatitis listed for liver transplantation in the US | Only reports epidemiology outcomes |
| Lim | 2023 | Natural history of NASH cirrhosis in liver transplant waitlist registrants | Only reports epidemiology outcomes |
| Lin | 2023 | Age and the relative importance of liver-related deaths in nonalcoholic fatty liver disease | Only reports epidemiology outcomes |
| Lombardi | 2024 | Comparison of the severity of metabolic, liver and cardiovascular damage in NAFLD patients attending the hepatology clinic over the last three decades | Only reports epidemiology outcomes |
| Loomba | 2020 | Nonalcoholic fatty liver disease progression rates to cirrhosis and progression of cirrhosis to decompensation and mortality: a real world analysis of Medicare data | Only reports epidemiology outcomes |
| Loy | 2018 | Gender disparities in liver transplant candidates with nonalcoholic steatohepatitis | Only reports epidemiology outcomes |
| Luger | 2016 | Prevalence of Liver Fibrosis and its Association with Non-invasive Fibrosis and Metabolic Markers in Morbidly Obese Patients with Vitamin D Deficiency | Only reports NITs not listed in PICOS |
| Mann | 2020 | Hospital admission with non-alcoholic fatty liver disease is associated with increased all-cause mortality independent of cardiovascular risk factors | Only reports epidemiology outcomes |
| Masarone | 2021 | Untargeted metabolomics as a diagnostic tool in NAFLD: discrimination of steatosis, steatohepatitis and cirrhosis | 20 or fewer patients |
| Matsushita | 2014 | Investigation of ornithine carbamoyltransferase as a biomarker of liver cirrhosis | Only reports epidemiology outcomes |
| McPherson | 2014 | Serum immunoglobulin levels predict fibrosis in patients with non-alcoholic fatty liver disease | Clinical study with 50 or fewer patients |
| Meroni | 2022 | Low Lipoprotein(a) Levels Predict Hepatic Fibrosis in Patients With Nonalcoholic Fatty Liver Disease | Only reports NITs not listed in PICOS |
| Mikolasevic | 2020 | Screening for nonalcoholic fatty liver disease in patients with type 2 diabetes mellitus using transient elastography - a prospective, cross sectional study | Only reports epidemiology outcomes |
| Miller | 2023 | Progression to cirrhosis is similar among all ages in nonalcoholic fatty liver disease, but liver-related events increase with age | Only reports epidemiology outcomes |
| Minich | 2022 | Predictors of patient survival following liver transplant in non-alcoholic steatohepatitis: A systematic review and meta-analysis | SLR |
| Mittal | 2024 | A prospective study on the prevalence of at-risk MASH in patients with type 2 diabetes mellitus in the United States | Only reports epidemiology outcomes |
| Mohagheghi | 2020 | Changes in the distribution of etiologies of cirrhosis among patients referred for liver transplantation over 11 years in Iran | Only reports epidemiology outcomes |
| Molinari | 2021 | Portal vein thrombosis and renal dysfunction: a national comparative study of liver transplant recipients for NAFLD versus alcoholic cirrhosis | Only reports epidemiology outcomes |
| Nagel | 2022 | Suppressed serological vitamin A in patients with liver cirrhosis is associated with impaired liver function and clinical detoriation | Only reports epidemiology outcomes |
| Natarajan | 2020 | Risk of Cirrhosis and Hepatocellular Cancer in Patients With NAFLD and Normal Liver Enzymes | Only reports NITs not listed in PICOS |
| Ng | 2023 | Mortality Outcomes by Fibrosis Stage in Nonalcoholic Fatty Liver Disease: A Systematic Review and Meta-analysis | SLR |
| Nguyen | 2019 | Rising Inpatient Encounters and Economic Burden for Patients with Nonalcoholic Fatty Liver Disease in the USA | Only reports epidemiology outcomes |
| Nguyen | 2023 | Differential Mortality Outcomes in Real-world Patients with Lean, Nonobese, and Obese Nonalcoholic Fatty Liver Disease | Only reports economic outcomes (other than healthcare costs/HCRU/liver transplant rates) |
| Nikitin | 2020 | Liver cirrhosis as the outcome of non-alcoholic fatty liver disease associated with PNPLA3 Gene RS738409 polymorphism | Only reports epidemiology outcomes |
| Nilsson | 2019 | Clinical course and mortality by etiology of liver cirrhosis in Sweden: a population based, long-term follow-up study of 1317 patients | Only reports epidemiology outcomes |
| Nogami | 2019 | Assessment of 10-year changes in liver stiffness using vibration-controlled transient elastography in non-alcoholic fatty liver disease | 20 or fewer patients |
| Nyberg | 2021 | The Natural History of NAFLD, a Community-Based Study at a Large Health Care Delivery System in the United States | Only reports epidemiology outcomes |
| O'Beirne | 2023 | Diabetes mellitus and the progression of non-alcoholic fatty liver disease to decompensated cirrhosis: a retrospective cohort study | Only reports epidemiology outcomes |
| Oh | 2020 | A Universal Gut-Microbiome-Derived Signature Predicts Cirrhosis | Clinical study with 50 or fewer patients |
| Onnerhag | 2014 | Increased risk of cirrhosis and hepatocellular cancer during long-term follow-up of patients with biopsy-proven NAFLD | 20 or fewer patients |
| Orci | 2022 | Incidence of Hepatocellular Carcinoma in Patients With Nonalcoholic Fatty Liver Disease: A Systematic Review, Meta-analysis, and Meta-regression | SLR |
| Orman | 2019 | Trends in Characteristics, Mortality, and Other Outcomes of Patients With Newly Diagnosed Cirrhosis | Only reports epidemiology outcomes |
| Panikar | 2024 | Prevalence and Association of Risk Factors According to Liver Steatosis and Fibrosis Stages among Nonalcoholic Fatty Liver Disease Patients with Type 2 Diabetes Mellitus in India: A Cross-sectional Study | Only reports epidemiology outcomes |
| Parikh | 2019 | Projected increase in obesity and non-alcoholic-steatohepatitis-related liver transplantation waitlist additions in the United States | Only reports epidemiology outcomes |
| Park | 2019 | Nonalcoholic fatty liver disease increases risk of incident advanced chronic kidney disease: a propensity-matched cohort study | Only reports epidemiology outcomes |
| Parra | 2024 | Prevalence of post-liver transplant complications and NASH-related cirrhosis in postmenopausal women | Only reports epidemiology outcomes |
| Parvataneni | 2024 | A comprehensive evaluation of emergency department utilization by patients with cirrhosis | Only reports epidemiology outcomes |
| Patel | 2018 | Coronary artery disease in decompensated patients undergoing liver transplantation evaluation | Only reports epidemiology outcomes |
| Patel | 2018 | Risk factors for biopsy-proven advanced non-alcoholic fatty liver disease in the Veterans Health Administration | Only reports epidemiology outcomes |
| Patel | 2021 | Progression to Cirrhosis Leads to Improvement in Atherogenic Milieu | Only reports epidemiology outcomes |
| Paternostro | 2021 | Prevalence of anti-Hepatitis E antibodies and impact on disease severity in non-alcoholic fatty liver disease | Only reports epidemiology outcomes |
| Paternostro | 2022 | The prognostic value of HVPG-response to non-selective beta-blockers in patients with NASH cirrhosis and varices | Clinical study with 50 or fewer patients |
| Pavlides | 2017 | Multiparametric magnetic resonance imaging for the assessment of non-alcoholic fatty liver disease severity | 20 or fewer patients |
| Petta | 2021 | Monitoring Occurrence of Liver-Related Events and Survival by Transient Elastography in Patients With Nonalcoholic Fatty Liver Disease and Compensated Advanced Chronic Liver Disease | Only reports NITs not listed in PICOS |
| Piscaglia | 2016 | Clinical patterns of hepatocellular carcinoma in nonalcoholic fatty liver disease: A multicenter prospective study | Only reports epidemiology outcomes |
| Pons | 2021 | Noninvasive Diagnosis of Portal Hypertension in Patients With Compensated Advanced Chronic Liver Disease | Only reports epidemiology outcomes |
| Popescu | 2016 | Non-alcoholic fatty liver disease - clinical and histopathological aspects | 20 or fewer patients |
| Qu | 2021 | Diagnostic Performance of FibroTouch Ultrasound Attenuation Parameter and Liver Stiffness Measurement in Assessing Hepatic Steatosis and Fibrosis in Patients With Nonalcoholic Fatty Liver Disease | 20 or fewer patients |
| Rashu | 2021 | Referral patterns for patients with nonalcoholic fatty liver disease | 20 or fewer patients |
| Rastogi | 2022 | Non-alcoholic fatty liver disease (NAFLD) in lean individuals - Single centre large cohort clinicopathologic and immunophenotypic study | Only reports epidemiology outcomes |
| Reig | 2019 | Should Patients With NAFLD/NASH Be Surveyed for HCC? | SLR |
| Rivas | 2021 | Plasminogen activator inhibitor is significantly elevated in liver transplant recipients with decompensated NASH cirrhosis | Only reports epidemiology outcomes |
| Sakai | 2021 | Clinical trial of autologous adipose tissue-derived regenerative (stem) cells therapy for exploration of its safety and efficacy | 20 or fewer patients |
| Samala | 2024 | Non-Hispanic Black Persons With Nonalcoholic Fatty Liver Disease Have Lower Rates of Advanced Fibrosis, Cirrhosis, and Liver-Related Events Even After Controlling for Clinical Risk Factors and PNPLA3 Genotype | Only reports epidemiology outcomes |
| Sanyal | 2019 | The Natural History of Advanced Fibrosis Due to Nonalcoholic Steatohepatitis: Data From the Simtuzumab Trials | Only reports epidemiology outcomes |
| Sanyal | 2021 | Prospective Study of Outcomes in Adults with Nonalcoholic Fatty Liver Disease | Only reports epidemiology outcomes |
| Selvaraj | 2021 | Diagnostic accuracy of elastography and magnetic resonance imaging in patients with NAFLD: A systematic review and meta-analysis | SLR |
| Setiawan | 2016 | Prevalence of chronic liver disease and cirrhosis by underlying cause in understudied ethnic groups: The multiethnic cohort | Only reports epidemiology outcomes |
| Shaheen | 2021 | Impact of major depression and antidepressant use on alcoholic and non-alcoholic fatty liver disease: A population-based study | Only reports epidemiology outcomes |
| Sharma | 2016 | A study on the etiology of cirrhosis of liver in adults living in the Hills of Himachal Pradesh, India | 20 or fewer patients |
| Sharpton | 2022 | Gut metagenome-derived signature predicts hepatic decompensation and mortality in NAFLD-related cirrhosis | Clinical study with 50 or fewer patients |
| Shelley | 2023 | Clinical characteristics and management of patients with nonalcoholic steatohepatitis in a real-world setting: analysis of the Ipsos NASH therapy monitor database | Only reports epidemiology outcomes |
| Shirazi | 2020 | Nonalcoholic Steatohepatitis Becomes the Leading Indication for Liver Transplant Registrants Among US Adults Born Between 1945 and 1965 | Only reports epidemiology outcomes |
| Siddiqui | 2015 | Severity of nonalcoholic fatty liver disease and progression to cirrhosis are associated with atherogenic lipoprotein profile | 20 or fewer patients |
| Simon | 2021 | Cancer Risk in Patients With Biopsy-Confirmed Nonalcoholic Fatty Liver Disease: A Population-Based Cohort Study | Only reports epidemiology outcomes |
| Simon | 2021 | Mortality in biopsy-confirmed nonalcoholic fatty liver disease: Results from a nationwide cohort | Only reports epidemiology outcomes |
| Simon | 2022 | Non-alcoholic fatty liver disease and incident major adverse cardiovascular events: results from a nationwide histology cohort | Only reports epidemiology outcomes |
| Simon | 2023 | Progression of non-alcoholic fatty liver disease and long-term outcomes: A nationwide paired liver biopsy cohort study | Only reports epidemiology outcomes |
| Singal | 2022 | Healthcare burden and outcomes of hepatorenal syndrome among cirrhosis-related hospitalisations in the US | Only reports economic outcomes (other than healthcare costs/HCRU/liver transplant rates) |
| Singh | 2024 | Do Patients With NASH-related Cirrhosis Have Better Overall Survival Compared With Other Etiologies of Cirrhosis? A Population-based Study | Only reports epidemiology outcomes |
| Skladany | 2021 | Frailty in Nonalcoholic Fatty Liver Cirrhosis: A Comparison with Alcoholic Cirrhosis, Risk Patterns, and Impact on Prognosis | Only reports epidemiology outcomes |
| Solanki | 2023 | A case-cohort study of left ventricular diastolic dysfunction in patients with cirrhosis: the liver-heart axis | Only reports epidemiology outcomes |
| Soresi | 2020 | The Prevalence of NAFLD and Fibrosis in Bariatric Surgery Patients and the Reliability of Noninvasive Diagnostic Methods | 20 or fewer patients |
| Stender | 2017 | Adiposity amplifies the genetic risk of fatty liver disease conferred by multiple loci | Only reports epidemiology outcomes |
| Stine | 2015 | Increased risk of portal vein thrombosis in patients with cirrhosis due to nonalcoholic steatohepatitis | Only reports epidemiology outcomes |
| Stine | 2018 | Increased risk of venous thromboembolism in hospitalized patients with cirrhosis due to non-alcoholic steatohepatitis original-contribution | Only reports epidemiology outcomes |
| Subasi | 2015 | Comparison of noninvasive scores for the detection of advanced fibrosis in patients with nonalcoholic fatty liver disease | 20 or fewer patients |
| Sun | 2015 | Nonalcoholic Cirrhosis Increased Risk of Digestive Tract Malignancies: A Population-Based Cohort Study | Only reports epidemiology outcomes |
| Swain | 2020 | Burden of nonalcoholic fatty liver disease in Canada, 2019-2030: a modelling study | Only reports epidemiology outcomes |
| Sydor | 2020 | Altered Microbiota Diversity and Bile Acid Signaling in Cirrhotic and Noncirrhotic NASH-HCC | Only reports epidemiology outcomes |
| Takahashi | 2023 | Association of Serum Albumin Levels and Long-Term Prognosis in Patients with Biopsy-Confirmed Nonalcoholic Fatty Liver Disease | Only reports epidemiology outcomes |
| Tampi | 2020 | Modelling the economic and clinical burden of non-alcoholic steatohepatitis in East Asia: Data from Hong Kong | Only reports epidemiology outcomes |
| Tarao | 2019 | Real impact of liver cirrhosis on the development of hepatocellular carcinoma in various liver diseases-meta-analytic assessment | SLR |
| Taylor | 2020 | Association Between Fibrosis Stage and Outcomes of Patients With Nonalcoholic Fatty Liver Disease: A Systematic Review and Meta-Analysis | SLR |
| Thanapirom | 2022 | Non-invasive tests for liver fibrosis assessment in patients with chronic liver diseases: a prospective study | 20 or fewer patients |
| Tokutsu | 2023 | Clinical characteristics in patients with non-alcoholic steatohepatitis in Japan: a case-control study using a 5-year large-scale claims database | Only reports epidemiology outcomes |
| Tran | 2024 | Updates in characteristics and survival rates of cirrhosis in a nationwide cohort of real-world U.S. patients, 2003-2021 | Only reports epidemiology outcomes |
| Turker | 2021 | Does the FT3-to-FT4 ratio easily predict the progression of NAFLD and NASH cirrhosis? | Only reports NITs not listed in PICOS |
| Unger | 2017 | The post-transplant course of patients undergoing liver transplantation for nonalcoholic steatohepatitis versus cryptogenic cirrhosis: A retrospective case-control study | 20 or fewer patients |
| Valery | 2021 | Changing prevalence of aetiological factors and comorbidities among Australians hospitalised for cirrhosis | Only reports epidemiology outcomes |
| Valery | 2024 | High prevalence of diabetes among young First Nations Peoples with metabolic dysfunction-associated steatotic liver disease: a population-based study in Australia | Only reports epidemiology outcomes |
| van den Berg | 2018 | Liver transplantation for NASH cirrhosis is not performed at the expense of major post-operative morbidity | Clinical study with 50 or fewer patients |
| Vatansever | 2019 | The evaluation of complications and mortality in non-alcoholic steatohepatitis-related cirrhosis | Only reports epidemiology outcomes |
| Vilar-Gomez | 2018 | Fibrosis Severity as a Determinant of Cause-Specific Mortality in Patients With Advanced Nonalcoholic Fatty Liver Disease: A Multi-National Cohort Study | Only reports epidemiology outcomes |
| Vorobioff | 2020 | A Latin American survey on demographic aspects of hospitalized, decompensated cirrhotic patients and the resources for their management | Only reports epidemiology outcomes |
| Wang | 2023 | Cause-specific mortality among patients with cirrhosis in a population-based cohort study in Ontario (2000-2017) | Only reports epidemiology outcomes |
| Wang | 2023 | Impacts of bariatric surgery on adverse liver outcomes: a systematic review and meta-analysis | SLR |
| Wang | 2024 | EUS-guided liver palpation as a screening tool for advanced fibrosis and cirrhosis in patients with suspected metabolic dysfunction-associated steatotic liver disease: a pilot study | Only reports epidemiology outcomes |
| Weinmann | 2014 | Trends in epidemiology, treatment, and survival of hepatocellular carcinoma patients between 1998 and 2009: An analysis of 1066 cases of a german HCC registry | Only reports epidemiology outcomes |
| Wijarnpreecha | 2021 | Association between sarcopenic obesity and nonalcoholic fatty liver disease and fibrosis detected by fibroscan | Only reports epidemiology outcomes |
| Wijarnpreecha | 2023 | Higher mortality among lean patients with non-alcoholic fatty liver disease despite fewer metabolic comorbidities | Only reports epidemiology outcomes |
| Wong | 2019 | Increasing metabolic co-morbidities are associated with higher risk of advanced fibrosis in nonalcoholic steatohepatitis | Only reports epidemiology outcomes |
| Wong | 2020 | Geographic regions with high prevalence of nonalcoholic steatohepatitis-related hepatic fibrosis are also observed to demonstrate high prevalence of metabolic disease risk factors and low consumption of fruits and vegetables | Only reports epidemiology outcomes |
| Wu | 2023 | Trends in the incidence of cirrhosis in global from 1990 to 2019: A joinpoint and age-period-cohort analysis | Only reports epidemiology outcomes |
| Wu | 2024 | Intake of the different types of dairy products, genetic predisposition, and the risks of nonalcoholic fatty liver disease and cirrhosis: a prospective cohort study | Only reports epidemiology outcomes |
| Xanthakos | 2015 | High Prevalence of Nonalcoholic Fatty Liver Disease in Adolescents Undergoing Bariatric Surgery | Only reports epidemiology outcomes |
| Xie | 2022 | Correlation analysis of metabolic characteristics and the risk of metabolic-associated fatty liver disease - related hepatocellular carcinoma | Only reports epidemiology outcomes |
| Xiong | 2015 | Non-alcoholic steatohepatitis-related liver cirrhosis is increasing in China: a ten-year retrospective study | Only reports epidemiology outcomes |
| Xu | 2023 | Performance of FibroScan in grading steatosis and fibrosis in patients with nonalcoholic fatty liver disease: A meta-analysis | SLR |
| Yang | 2020 | Diabetes Is Associated With Increased Risk of Hepatocellular Carcinoma in Patients With Cirrhosis From Nonalcoholic Fatty Liver Disease | Only reports epidemiology outcomes |
| Ye | 2020 | Global prevalence, incidence, and outcomes of non-obese or lean non-alcoholic fatty liver disease: a systematic review and meta-analysis | SLR |
| Yeoh | 2024 | Incidence of Cirrhosis and Hepatocellular Carcinoma Among Veterans With Noncirrhotic Metabolic Dysfunction-associated Fatty Liver Disease | Only reports epidemiology outcomes |
| Yilmaz | 2019 | Growing burden of nonalcoholic fatty liver disease in Turkey: A single-center experience | 20 or fewer patients |
| Yong | 2023 | Outcomes of Nonalcoholic Steatohepatitis After Liver Transplantation: An Updated Meta-Analysis and Systematic Review | SLR |
| Younossi | 2022 | The burden of non-alcoholic steatohepatitis: A systematic review of health-related quality of life and patient-reported outcomes | SLR |
| Younossi | 2023 | The Potential Role of Fatigue in Identifying Patients With NASH and Advanced Fibrosis Who Experience Disease Progression | Only reports the fatigue domain of the CLDQ-NASH |
| Younossi | 2024 | Estimating the economic impact of comorbidities in patients with MASH and defining high-cost burden in patients with noncirrhotic MASH | Only reports economic outcomes (other than healthcare costs/HCRU/liver transplant rates) |
| Younossi | 2024 | Liver histology is associated with long-term clinical outcomes in patients with metabolic dysfunction-associated steatohepatitis | Only reports epidemiology outcomes |
| Zambrano-Huailla | 2020 | Diagnostic performance of three non-invasive fibrosis scores (Hepamet, FIB-4, NAFLD fibrosis score) in NAFLD patients from a mixed Latin American population | 20 or fewer patients |
| Zheng | 2022 | The role of dietary factors in nonalcoholic fatty liver disease to hepatocellular carcinoma progression: A systematic review | SLR |
| Zhou | 2022 | Diagnosis of steatohepatitis and fibrosis in biopsy-proven nonalcoholic fatty liver diseases: Including two-dimension real-time shear wave elastography and noninvasive fibrotic biomarker scores | 20 or fewer patients |

Expansions of abbreviations are only provided for those used in the column ‛Reasons excluding from data extraction (if applicable)’.

Abbreviations: CLDQ-NASH, Chronic Liver Disease Questionnaire-Non-Alcoholic Steatohepatitis; HCRU, healthcare resource utilisation; NIT, non-invasive test; PICOS, population, intervention, comparator, outcomes; SLR, systematic literature review.

## Table S5. List of 74 eligible studies with data extracted for clinical outcomes and use of NITs

| **Author** | **Year** | **Title** | **Geography** | **Design** | **Perspective** | **Study period** | **Outcome(s)** |
| --- | --- | --- | --- | --- | --- | --- | --- |
| Andrade | 2022 | Risk predictors of advanced hepatic fibrosis in patients with nonalcoholic fatty liver disease - a survey in a university hospital in Brazil | Brazil | Observational | Retrospective | 2000–2018 | NITs |
| Are | 2021 | Enhanced Liver Fibrosis Score Can Be Used to Predict Liver-Related Events in Patients With Nonalcoholic Steatohepatitis and Compensated Cirrhosis | USA | RCT | Prospective | NR | NITs |
| Bertot | 2018 | Diabetes impacts prediction of cirrhosis and prognosis by non-invasive fibrosis models in non-alcoholic fatty liver disease | Australia | Observational | Unclear | 2006–2015 | NITs |
| Brandman | 2022 | Comparison of clinical prediction rules for ruling out cirrhosis in nonalcoholic fatty liver disease (NAFLD) | USA and UK | Observational | Prospective | US cohort: 2004–2008; 2009–2015  UK cohort: NR | NITs |
| Bril | 2024 | Noninvasive tests to identify liver fibrosis in metabolic dysfunction-associated steatotic liver disease are affected by race | USA | Observational | Unclear | 2019–2020 | NITs |
| Calzadilla-Bertot | 2021 | ABIDE: An Accurate Predictive Model of Liver Decompensation in Patients With Nonalcoholic Fatty Liver-Related Cirrhosis | USA | Data derived from models | Retrospective | Derivation cohort: 1995–2013  Validation cohort: 2004–2016 | NITs |
| Chang | 2023 | Machine learning models are superior to noninvasive tests in identifying clinically significant stages of NAFLD and NAFLD-related cirrhosis | USA | Observational | Retrospective | 2015–2020 | NITs |
| Eddowes | 2019 | Accuracy of FibroScan Controlled Attenuation Parameter and Liver Stiffness Measurement in Assessing Steatosis and Fibrosis in Patients With Nonalcoholic Fatty Liver Disease | UK | Observational | Prospective | 2014–2017 | NITs |
| Gerhardt | 2020 | Biopsy rate and nonalcoholic steatohepatitis (NASH) in patients with nonalcoholic fatty liver disease (NAFLD) | Germany | Observational | Retrospective | 2013–2018 | NITs |
| Gordon | 2020 | Health Care Use and Costs Among Patients With Nonalcoholic Steatohepatitis With Advanced Fibrosis Using the Fibrosis-4 Score | USA | Observational | Retrospective | 2008–2016 | NITs |
| Hagstrom | 2020 | Ability of Noninvasive Scoring Systems to Identify Individuals in the Population at Risk for Severe Liver Disease | Sweden | Observational | Retrospective | 1985–1996 | NITs |
| Imajo | 2022 | Direct Comparison of US and MR Elastography for Staging Liver Fibrosis in Patients With Nonalcoholic Fatty Liver Disease | Japan | Observational | Prospective | Enrolment period: 2018–2020 | NITs |
| Kaplan | 2024 | Clinical and genetic risk factors for progressive fibrosis in metabolic dysfunction-associated steatotic liver disease | USA | Observational | Retrospective | 2002–2021 | NITs |
| Karlas | 2018 | Impact of controlled attenuation parameter on detecting fibrosis using liver stiffness measurement | NR | Secondary analysis of meta-analysis | Retrospective | NR | NITs |
| Koch | 2024 | H2FPEF Scores Are Increased in Patients with NASH Cirrhosis and Are Associated with Post-liver Transplant Heart Failure | USA | Observational | Retrospective | 2010–2018 | NITs |
| Liu | 2021 | A prospective 5-year study on the use of transient elastography to monitor the improvement of non-alcoholic fatty liver disease following bariatric surgery | China | Observational | Prospective | 2011–2013 | NITs |
| Loomba | 2023 | Liver stiffness thresholds to predict disease progression and clinical outcomes in bridging fibrosis and cirrhosis | Multiple countries worldwide | Pooled analysis | Retrospective | NR | NITs |
| Matsumoto | 2021 | Ultrasonographic grayscale findings related to fibrosis in patients with non-alcoholic fatty liver disease: comparison with transient elastography and Fib-4 index | Japan | Observational | Retrospective | 2015–2020 | NITs |
| Mozes | 2024 | Diagnostic accuracy of non-invasive tests to screen for at-risk MASH-An individual participant data meta-analysis | NR | Meta-analysis | Retrospective | NR | NITs |
| Noureddin | 2023 | Increased accuracy in identifying NAFLD with advanced fibrosis and cirrhosis: independent validation of the Agile 3+ and 4 scores | USA | Observational | Retrospective | 2014–2021 | NITs |
| Sanyal | 2023 | Diagnostic performance of circulating biomarkers for non-alcoholic steatohepatitis | USA | Observational | Unclear | NR | NITs |
| Seko | 2023 | Diagnostic accuracy of enhanced liver fibrosis test for nonalcoholic steatohepatitis-related fibrosis: Multicenter study | Japan | Observational | Unclear | 1990–2020 | NITs |
| Shabbir | 2024 | Role of Alanine Transaminase and Transient Elastography in Categorising Nonalcoholic Fatty Liver Disease Subgroups | Pakistan | Observational | Unclear | 2022 | NITs |
| Srivastava | 2019 | Prospective evaluation of a primary care referral pathway for patients with non-alcoholic fatty liver disease | UK | Observational | Prospective | 2014–2016 | NITs |
| Tang | 2023 | N-terminal propeptide of type 3 collagen-based sequential algorithm can identify high-risk steatohepatitis and fibrosis in MAFLD | China | Observational | Unclear | Derivation cohort: 2017–2020  Validation cohort: 2021 | NITs |
| Taru | 2024 | FAST and Agile-the MASLD drift: Validation of Agile 3+, Agile 4 and FAST scores in 246 biopsy-proven NAFLD patients meeting MASLD criteria of prevalent Caucasian origin | Romania | Observational | Retrospective | 2007–2023 | NITs |
| Vieira Barbosa | 2022 | Fibrosis-4 Index as an Independent Predictor of Mortality and Liver-Related Outcomes in NAFLD | USA | Observational | Retrospective | 2015–2019 | NITs |
| Wang | 2024 | EUS-guided liver palpation as a screening tool for advanced fibrosis and cirrhosis in patients with suspected metabolic dysfunction-associated steatotic liver disease: a pilot study | USA | Observational | Prospective | 2021–2023 | NITs |
| Wong | 2019 | Unified interpretation of liver stiffness measurement by M and XL probes in non-alcoholic fatty liver disease | France and China | Observational | Prospective | 2009–2017 | NITs |
| Younossi | 2019 | Reduced Patient-Reported Outcome Scores Associate With Level of Fibrosis in Patients With Nonalcoholic Steatohepatitis | Multiple countries worldwide | Pooled analysis | Retrospective | 2017–2018 | NITs |
| Younossi | 2021 | Improvements of Fibrosis and Disease Activity Are Associated With Improvement of Patient-Reported Outcomes in Patients With Advanced Fibrosis Due to Nonalcoholic Steatohepatitis | Multiple countries worldwide | Pooled analysis | Retrospective | NR | NITs |
| Alkhouri | 2016 | Liver transplantation for nonalcoholic steatohepatitis in young patients | USA | Observational | Unclear | 1987–2012 | Clinical |
| Axley | 2019 | NASH Is the Most Rapidly Growing Etiology for Acute-on-Chronic Liver Failure-Related Hospitalization and Disease Burden in the United States: A Population-Based Study | USA | Observational | Retrospective | 2006–2014 | Clinical |
| Becker | 2024 | Disparities in Liver Transplantation for Nonalcoholic Steatohepatitis in Women | USA | Observational | Retrospective | 1997–2021 | Clinical |
| Chu | 2024 | Underrecognition and Suboptimal Quality of Care for Nonalcoholic Fatty Liver Disease Cirrhosis in Primary Care Patients with Diabetes Mellitus | USA | Observational | Unclear | 2017–2020 | Clinical |
| Doycheva | 2018 | Nonalcoholic Steatohepatitis is the Most Rapidly Increasing Indication for Liver Transplantation in Young Adults in the United States | USA | Observational | Retrospective | 2002–2012 | Clinical |
| Eshraghian | 2022 | Nonalcoholic Steatohepatitis Is the Most Rapidly Growing Indication for Liver Transplantation in Iranian Patients | Iran | Observational | Retrospective | 1993–2017 | Clinical |
| Ferrarese | 2022 | Nash Up, Virus Down: How the Waiting List Is Changing for Liver Transplantation: A Single Center Experience from Italy | Italy | Observational | Retrospective | 2006–2020 | Clinical |
| Frenette | 2021 | Emricasan to prevent new decompensation in patients with NASH-related decompensated cirrhosis | USA | RCT | Prospective | 2017–2019 | Clinical |
| Garcia-Tsao | 2020 | Randomized placebo-controlled trial of emricasan for non-alcoholic steatohepatitis-related cirrhosis with severe portal hypertension | USA and Europe | RCT | Prospective | 2016–2018 | Clinical |
| Harrison | 2018 | Simtuzumab Is Ineffective for Patients With Bridging Fibrosis or Compensated Cirrhosis Caused by Nonalcoholic Steatohepatitis | Multiple countries worldwide | RCT | Prospective | 2013–2014 | Clinical |
| Harrison | 2020 | Selonsertib for patients with bridging fibrosis or compensated cirrhosis due to NASH: Results from randomized phase III STELLAR trials | Multiple countries worldwide | RCT | Prospective | 2017–2019^a^ | Clinical |
| Karnam | 2023 | Impact of living donor liver transplantation on long-term cardiometabolic and graft outcomes in cirrhosis due to nonalcoholic steatohepatitis | Canada | Observational | Retrospective | 2002–2018 | Clinical |
| Kern | 2019 | High incidence of hepatocellular carcinoma and postoperative complications in patients with nonalcoholic steatohepatitis as a primary indication for deceased liver transplantation | Austria | Observational | Retrospective | 2002–2012 | Clinical |
| Lee | 2024 | Etiology-Specific Effects of Impaired Functional Status on Liver Transplant Outcomes | USA | Observational | Retrospective | 2005–2019 | Clinical |
| Loomba | 2023 | Semaglutide 2.4 mg once weekly in patients with non-alcoholic steatohepatitis-related cirrhosis: a randomised, placebo-controlled phase 2 trial | USA and Europe | RCT | Prospective | 2019–2021 | Clinical |
| Nagai | 2019 | Increased Risk of Death in First Year After Liver Transplantation Among Patients With Nonalcoholic Steatohepatitis vs Liver Disease of Other Etiologies | USA | Observational | Retrospective | 2008–2018 | Clinical |
| Oladunjoye | 2021 | A Retrospective Study of Hospitalizations in the USA: Proportion of Hospitalizations With Non-Alcoholic Fatty Liver Disease in Non-Obese Population | USA | Observational | Retrospective | 2010–2014 | Clinical |
| Paik | 2023 | Non-alcoholic fatty liver disease is associated with greater risk of 30-day hospital readmission in the United States (U.S.) | USA | Observational | Prospective | 2010–2017 | Clinical |
| Qayed | 2023 | Characteristics and Outcomes of Black and White Patients Hospitalized with Nonalcoholic Steatohepatitis: A Nationwide Analysis | USA | Observational | Retrospective | 2016–2018 | Clinical |
| Qazi-Arisar | 2022 | Divergent trajectories of lean vs obese non-alcoholic steatohepatitis patients from listing to post-transplant: A retrospective cohort study | Canada | Observational | Retrospective | 2012–2020 | Clinical |
| Reznicek | 2023 | Outcomes of Liver Transplantation in Patients With Preexisting Coronary Artery Disease | USA | Observational | Retrospective | 2013–2018 | Clinical |
| Rinella | 2023 | Factors Impacting Survival in Those Transplanted for NASH Cirrhosis: Data From the NailNASH Consortium | USA | Observational | Retrospective | 1997–2017 | Clinical |
| Rudolfsen | 2024 | Societal costs and survival of patients with biopsy-verified non-alcoholic steatohepatitis: Danish nationwide register-based study | Denmark | Observational | Prospective | 1997–2021 | Clinical |
| Salman | 2021 | Impact of laparoscopic sleeve gastrectomy on fibrosis stage in patients with child-A NASH-related cirrhosis | Egypt | Single arm interventional trial | Prospective | 2012–2016 | Clinical |
| Thuluvath | 2019 | Poor Survival after Retransplantation in NASH Cirrhosis | USA | Observational | Retrospective | 2002–2016 | Clinical |
| Younossi | 2021 | Nonalcoholic Steatohepatitis Is the Most Rapidly Increasing Indication for Liver Transplantation in the United States | USA | Observational | Retrospective | 2002–2019^a^ | Clinical |
| Zou | 2020 | A Nationwide Study of Inpatient Admissions, Mortality, and Costs for Patients with Cirrhosis from 2005 to 2015 in the USA | USA | Observational | Retrospective | 2005–2015 | Clinical |
| Cholankeril | 2023 | Longitudinal changes in fibrosis markers are associated with risk of cirrhosis and hepatocellular carcinoma in non-alcoholic fatty liver disease | USA | Observational | Retrospective | 2004–2008 | Clinical and NITs |
| Khajehahmadi | 2024 | Increasing Prevalence and High Survival Rate of Liver Transplanted Patients with NASH and PSC Cirrhosis | Iran | Observational | Retrospective | 2001–2018 | Clinical and NITs |
| Mozes | 2023 | Performance of non-invasive tests and histology for the prediction of clinical outcomes in patients with non-alcoholic fatty liver disease: an individual participant data meta-analysis | NR | Meta-analysis | Retrospective | 2003–2021 | Clinical and NITs |
| Parrish | 2019 | The Changing Face of Liver Transplantation in the United States: The Effect of HCV Antiviral Eras on Transplantation Trends and Outcomes | USA | Observational | Retrospective | 2003–2017 | Clinical and NITs |
| Pinyopornpanish | 2021 | Chemopreventive Effect of Statin on Hepatocellular Carcinoma in Patients With Nonalcoholic Steatohepatitis Cirrhosis | USA | Observational | Retrospective | 2002–2016 | Clinical and NITs |
| Quillin | 2014 | Increasing prevalence of nonalcoholic steatohepatitis as an indication for liver transplantation | USA | Observational | Retrospective | 2000–2012 | Clinical and NITs |
| Rinella | 2024 | A randomized, double-blind, placebo-controlled trial of aldafermin in patients with NASH and compensated cirrhosis | Multiple countries worldwide | RCT | Prospective | 2020–2022 | Clinical and NITs |
| Sanyal | 2022 | Cirrhosis regression is associated with improved clinical outcomes in patients with nonalcoholic steatohepatitis | NR | RCT | Prospective | Stimtuzmab RCT: 2013–2014 Selonsertib RCT: 2017–2018 | Clinical and NITs |
| Tan | 2021 | Poor Outcomes of Cirrhosis due to Nonalcoholic Steatohepatitis Compared With Hepatitis B After Decompensation With Ascites | Singapore | Observational | Retrospective | 2004–2015 | Clinical and NITs |
| Thuluvath | 2019 | Waiting List Mortality and Transplant Rates for NASH Cirrhosis When Compared With Cryptogenic, Alcoholic, or AIH Cirrhosis | USA | Observational | Retrospective | 2002–2016 | Clinical and NITs |
| Vijayaraghavan | 2023 | Intragastric Balloon in Obese Compensated Nonalcoholic Steatohepatitis Cirrhosis Patients Is Safe and Achieves Significant Weight Reduction at 6-Months | India | Observational | Prospective | 2017–2020 | Clinical and NITs |
| Vilar-Gomez | 2021 | Type 2 Diabetes and Metformin Use Associate With Outcomes of Patients With Nonalcoholic Steatohepatitis-Related, Child-Pugh A Cirrhosis | Multiple countries worldwide | Observational | Retrospective | 1995–2016 | Clinical and NITs |
| Villeret | 2022 | Liver transplantation for NAFLD cirrhosis: Age and recent coronary angioplasty are major determinants of survival | Europe | Observational | Retrospective | 2000–2019 | Clinical and NITs |
| Villeret | 2023 | Inevitability of disease recurrence after liver transplantation for NAFLD cirrhosis | Europe | Observational | Retrospective | 2000–2019 | Clinical and NITs |
| Wang | 2022 | Serum Fibrosis Tests Guide Prognosis in Metabolic Dysfunction-Associated Fatty Liver Disease Patients Referred From Primary Care | Australia | Observational | Retrospective | Cohort 1: 2004–2018 Cohort 2: NR | Clinical and NITs |
| Younossi | 2021 | The Association of Histologic and Noninvasive Tests With Adverse Clinical and Patient-Reported Outcomes in Patients With Advanced Fibrosis Due to Nonalcoholic Steatohepatitis | Multiple countries worldwide | RCT | Prospective | 2012–2019 | Clinical and NITs |

^a^Study start and completion years were retrieved from ClinicalTrials.gov (accessed 25 July 2025).
Expansions of abbreviations are only provided for those used in the columns ‛Geography’, ‘Design’, ‘Perspective’, ‘Study period’, ‘Outcome(s)’.

Abbreviations: NIT, non-invasive test; NR, not reported; RCT, randomised controlled trial.

## Table S6. Post-liver transplant survival in MASH stage F4

| **Country** | **Patients with MASH stage F4** | | **Study**  **period** | **Post-liver transplant survival, %** | | |
| --- | --- | --- | --- | --- | --- | --- |
|  | Mean (SD) age, years  (unless otherwise specified) | Women, % |  | 1 year | 3 years | 5 years |
| **Austria [**[**2**](#_ENREF_2)**]** | 60 (7) | 23 | 2002–2012 | 93 | 79 | 72 |
| **France and Switzerland [**[**9**](#_ENREF_9)**]** | Median (IQR): 62 (57–66) | 30 | 2000–2019 | 89 | NR | 80 |
| **Iran [**[**50**](#_ENREF_50)**]** | 53 (9) | 29 | 1993–2017 | 87 | NR | 85 |
| **Italy [**[**51**](#_ENREF_51)**]** | 59 (9) | 19 | 2006–2020 | 86 | 77 | 73 |
| **USA [**[**52**](#_ENREF_52)**]** | 34 (7) | 48 | 1987–2012 | 89 | 84 | 78 |
| **USA [**[**53**](#_ENREF_53)**]** | 59 (8) | 48 | 1997–2017 | 93 | 88 | 83 |
| **USA [**[**54**](#_ENREF_54)**]** | 35 (5) | 45.6 | 2002–2012 | NR | NR | 76 |

Abbreviations: F4, fibrosis stage 4; IQR, interquartile range, MASH, metabolic dysfunction-associated steatohepatitis, NR, not reported; SD, standard deviation.

## Table S7. Studies reporting AUROCs of NITs for identifying cirrhosis in patients with MASH

|  | **Brandman 2022 [**[**46**](#_ENREF_46)**]** | **Imajo 2022 [**[**17**](#_ENREF_17)**]** | **Sanyal 2023 [**[**45**](#_ENREF_45)**]** | **Chang 2023 [**[**34**](#_ENREF_34)**]** | **Noureddin 2023 [**[**39**](#_ENREF_39)**]** | **Mozes 2024 [**[**25**](#_ENREF_25)**]** | **Bril 2024 [**[**33**](#_ENREF_33)**]** |
| --- | --- | --- | --- | --- | --- | --- | --- |
| Reference standard for diagnosing cirrhosis | Biopsy | Biopsy | Biopsy | Biopsy | Biopsy | Biopsy | VCTE^a^ |
| **Blood-based NITs, AUROC (95% CI)** | | | | | | | |
| APRI score | 0.79  (NR) | – | – | – | – | – | 0.65  (0.60–0.70) |
| AST:ALT ratio | 0.77  (NR) | – | – | – | – | – | – |
| BARD score | 0.76  (NR) | – | – | – | – | – | – |
| Bonacini score | 0.73^b^  (NR) | – | – | – | – | – | – |
| ELF | – | – | 0.86  (0.82–0.89) | – | – | – | – |
| FIB-4 score | 0.87  (NR) | 0.83  (0.75–0.89) | 0.81  (0.77–0.85) | 0.85  (0.81–0.88) | – | 0.81  (0.77–0.84) | 0.67  (0.62–0.72) |
| Lok index | 0.84^b^  (NR) | – | – | – | – | – | – |
| NFS | 0.89  (NR) | 0.85  (0.78–0.90) | – | – | – | 0.76  (0.72–0.79) | 0.81  (0.77–0.85) |
| NIS4 | – | – | 0.73  (0.68–0.76) | – | – | – | – |
| PRO-C3 | – | – | 0.73  (0.69–0.77) | – | – | – | – |
| **VCTE/imaging-based NITs, AUROC (95% CI)** | | | | | | | |
| FibroScan | – | – | – | 0.86  (0.82–0.89) | – | – | – |
| LSM by VCTE | – | 0.87  (0.81–0.92) | – | – | 0.83  (0.78–0.87) | 0.84  (0.82–0.87) | – |
| 2D-SWE | – | 0.89  (0.84–0.93) | – | – | – | – | – |
| MRE | – | 0.92  (0.87–0.96) | – | – | – | – | – |
| **Combinations of blood- and VCTE-based NITs, AUROC (95% CI)** | | | | | | | |
| Agile 4 | – | – | – | – | 0.85  (0.81–0.88) | – | – |
| FAST | – | – | – | – | – | 0.79  (0.76–0.82) | – |
| FibroMeter VCTE | **–** | – | 0.90  (0.84–0.95) | – | – | – | – |
| Random forest | 0.89  (NR) | – | – | 0.89  (0.86–0.92) | – | – | – |

^a^Cirrhosis was defined based on a liver stiffness measurement ≥13.6 kPa. ^b^Only 424 patients were analysed for the Bonacini score and Lok index.

Abbreviations: 2D-SWE, two-dimensional shear wave elastography; ALT, alanine transaminase; APRI, aspartate transaminase to platelet ratio index; AST, aspartate transaminase; AUROC, area under the receiver operator characteristic curve; BARD, body mass index AST/ALT ratio diabetes; CI, confidence interval; ELF, Enhanced Liver Fibrosis; FAST, FibroScan-AST; FIB-4, fibrosis-4 index; LSM, liver stiffness measurement; MASH, metabolic dysfunction-associated steatohepatitis; MRE, magnetic resonance elastography; NFS, non-alcoholic fatty liver disease fibrosis score; NIS4, non-alcoholic steatohepatitis index-4; NIT, non-invasive test; NR, not reported; PRO-C3, N-terminal pro-peptide cleaved from the procollagen of type III collagen; VCTE, vibration-controlled transient elastography.

# References

1. Parrish NF, Feurer ID, Matsuoka LK, Rega SA, Perri R, Alexopoulos SP, "The Changing Face of Liver Transplantation in the United States: The Effect of HCV Antiviral Eras on Transplantation Trends and Outcomes," *Transplantation Direct* 5, no. 3 (2019): e427.

2. Kern B, Feurstein B, Fritz J, et al., "High Incidence of Hepatocellular Carcinoma and Postoperative Complications in Patients With Nonalcoholic Steatohepatitis as a Primary Indication for Deceased Liver Transplantation," *European Journal of Gastroenterology & Hepatology* 31, no. 2 (2019): 205–210.

3. Qazi-Arisar FA, Uchila R, Chen C, et al., "Divergent Trajectories of Lean vs Obese Non-Alcoholic Steatohepatitis Patients from Listing to Post-Transplant: A Retrospective Cohort Study," *World Journal of Gastroenterology* 28, no. 26 (2022): 3218–3231.

4. Bertot LC, Jeffrey GP, de Boer B, et al., "Diabetes Impacts Prediction of Cirrhosis and Prognosis by Non-Invasive Fibrosis Models in Non-Alcoholic Fatty Liver Disease," *Liver International* 38, no. 10 (2018): 1793–1802.

5. Wang Z, Bertot LC, Jeffrey GP, et al., "Serum Fibrosis Tests Guide Prognosis in Metabolic Dysfunction-Associated Fatty Liver Disease Patients Referred From Primary Care," *Clinical Gastroenterology and Hepatology* 20, no. 9 (2022): 2041–2049 e2045.

6. Andrade TG, Xavier LCD, Souza FF, Araujo RC, "Risk Predictors of Advanced Hepatic Fibrosis in Patients With Nonalcoholic Fatty Liver Disease - a Survey in a University Hospital in Brazil," *Archives of Endocrinology and Metabolism* 66, no. 6 (2022): 823–830.

7. Liu SY, Wong VW, Wong SK, et al., "A Prospective 5-Year Study on the Use of Transient Elastography to Monitor the Improvement of Non-Alcoholic Fatty Liver Disease Following Bariatric Surgery," *Scientific Reports* 11, no. 1 (2021): 5416.

8. Tang LJ, Li G, Eslam M, et al., "N-Terminal Propeptide of Type 3 Collagen-Based Sequential Algorithm Can Identify High-Risk Steatohepatitis and Fibrosis in MAFLD," *Hepatology International* 17, no. 1 (2023): 190–201.

9. Villeret F, Dharancy S, Erard D, et al., "Liver Transplantation for NAFLD Cirrhosis: Age and Recent Coronary Angioplasty Are Major Determinants of Survival," *Liver International* 42, no. 11 (2022): 2428–2441.

10. Villeret F, Dharancy S, Erard D, et al., "Inevitability of Disease Recurrence after Liver Transplantation for NAFLD Cirrhosis," *JHEP Reports* 5, no. 3 (2023): 100668.

11. Wong VW, Irles M, Wong GL, et al., "Unified Interpretation of Liver Stiffness Measurement by M and Xl Probes in Non-Alcoholic Fatty Liver Disease," *Gut* 68, no. 11 (2019): 2057–2064.

12. Gerhardt F, Petroff D, Blank V, et al., "Biopsy Rate and Nonalcoholic Steatohepatitis (NASH) in Patients With Nonalcoholic Fatty Liver Disease (NAFLD)," *Scandinavian Journal of Gastroenterology* 55, no. 6 (2020): 706–711.

13. Vijayaraghavan R, Sarin SK, Bharadwaj A, et al., "Intragastric Balloon in Obese Compensated Nonalcoholic Steatohepatitis Cirrhosis Patients is Safe and Achieves Significant Weight Reduction at 6-Months," *Digestive Diseases and Sciences* 68, no. 3 (2023): 1035–1041.

14. Khajehahmadi Z, Nikeghbalian S, Roshanaei G, Mohagheghi S, "Increasing Prevalence and High Survival Rate of Liver Transplanted Patients With NASH and PSC Cirrhosis," *Archives of Iranian Medicine* 27, no. 1 (2024): 23–29.

15. Matsumoto N, Kumagawa M, Ogawa M, et al., "Ultrasonographic Grayscale Findings Related to Fibrosis in Patients With Non-Alcoholic Fatty Liver Disease: Comparison with Transient Elastography and Fib-4 Index," *Journal of Medical Ultrasonics* 48, no. 3 (2021): 323–333.

16. Seko Y, Takahashi H, Toyoda H, et al., "Diagnostic Accuracy of Enhanced Liver Fibrosis Test for Nonalcoholic Steatohepatitis-Related Fibrosis: Multicenter Study," *Hepatology Research* 53, no. 4 (2023): 312–321.

17. Imajo K, Honda Y, Kobayashi T, et al., "Direct Comparison of US and MR Elastography for Staging Liver Fibrosis in Patients With Nonalcoholic Fatty Liver Disease," *Clinical Gastroenterology and Hepatology* 20, no. 4 (2022): 908–917 e911.

18. Rinella ME, Lieu HD, Kowdley KV, et al., "A Randomized, Double-Blind, Placebo-Controlled Trial of Aldafermin in Patients With NASH and Compensated Cirrhosis," *Hepatology* 79, no. 3 (2024): 674–689.

19. Vilar-Gomez E, Calzadilla-Bertot L, Wong VW, et al., "Type 2 Diabetes and Metformin Use Associate With Outcomes of Patients With Nonalcoholic Steatohepatitis-Related, Child-Pugh A Cirrhosis," *Clinical Gastroenterology and Hepatology* 19, no. 1 (2021): 136–145 e136.

20. Younossi ZM, Stepanova M, Anstee QM, et al., "Reduced Patient-Reported Outcome Scores Associate With Level of Fibrosis in Patients With Nonalcoholic Steatohepatitis," *Clinical Gastroenterology and Hepatology* 17, no. 12 (2019): 2552–2560 e2510.

21. Younossi ZM, Anstee QM, Wai-Sun Wong V, et al., "The Association of Histologic and Noninvasive Tests With Adverse Clinical and Patient-Reported Outcomes in Patients With Advanced Fibrosis Due to Nonalcoholic Steatohepatitis," *Gastroenterology* 160, no. 5 (2021): 1608–1619 e1613.

22. Younossi ZM, Stepanova M, Noureddin M, et al., "Improvements of Fibrosis and Disease Activity Are Associated With Improvement of Patient-Reported Outcomes in Patients With Advanced Fibrosis Due to Nonalcoholic Steatohepatitis," *Hepatology Communications* 5, no. 7 (2021): 1201–1211.

23. Mozes FE, Lee JA, Vali Y, et al., "Performance of Non-Invasive Tests and Histology for the Prediction of Clinical Outcomes in Patients With Non-Alcoholic Fatty Liver Disease: An Individual Participant Data Meta-Analysis," *The Lancet Gastroenterology and Hepatology* 8, no. 8 (2023): 704–713.

24. Karlas T, Petroff D, Sasso M, et al., "Impact of Controlled Attenuation Parameter on Detecting Fibrosis Using Liver Stiffness Measurement," *Alimentary Pharmacology & Therapeutics* 47, no. 7 (2018): 989–1000.

25. Mozes FE, Lee JA, Vali Y, et al., "Diagnostic Accuracy of Non-Invasive Tests to Screen for At-Risk MASH-An Individual Participant Data Meta-Analysis," *Liver International* 44, no. 8 (2024): 1872–1885.

26. Sanyal AJ, Anstee QM, Trauner M, et al., "Cirrhosis Regression is Associated With Improved Clinical Outcomes in Patients With Nonalcoholic Steatohepatitis," *Hepatology* 75, no. 5 (2022): 1235–1246.

27. Shabbir A, Abbas Z, Khatoon A, Mirza T, "Role of Alanine Transaminase and Transient Elastography in Categorising Nonalcoholic Fatty Liver Disease Subgroups," *Journal of College of Physicians and Surgeons Pakistan* 34, no. 1 (2024): 22–26.

28. Taru MG, Tefas C, Neamti L, et al., "FAST and Agile-the MASLD Drift: Validation of Agile 3+, Agile 4 and FAST Scores in 246 Biopsy-Proven NAFLD Patients Meeting MASLD CRITERIA of Prevalent Caucasian Origin," *PLoS ONE* 19, no. 5 (2024): e0303971.

29. Tan HK, Teng MLP, Soh AYS, et al., "Poor Outcomes of Cirrhosis due to Nonalcoholic Steatohepatitis Compared With Hepatitis B After Decompensation With Ascites," *American Journal of Gastroenterology* 116, no. 7 (2021): 1437–1446.

30. Hagstrom H, Talback M, Andreasson A, Walldius G, Hammar N, "Ability of Noninvasive Scoring Systems to Identify Individuals in the Population at Risk for Severe Liver Disease," *Gastroenterology* 158, no. 1 (2020): 200–214.

31. Eddowes PJ, Sasso M, Allison M, et al., "Accuracy of FibroScan Controlled Attenuation Parameter and Liver Stiffness Measurement in Assessing Steatosis and Fibrosis in Patients With Nonalcoholic Fatty Liver Disease," *Gastroenterology* 156, no. 6 (2019): 1717–1730.

32. Srivastava A, Gailer R, Tanwar S, et al., "Prospective Evaluation of a Primary Care Referral Pathway for Patients With Non-Alcoholic Fatty Liver Disease," *Journal of Hepatology* 71, no. 2 (2019): 371–378.

33. Bril F, Gray M, "Noninvasive Tests to Identify Liver Fibrosis in Metabolic Dysfunction-Associated Steatotic Liver Disease Are Affected by Race," *Obesity (Silver Spring)* 32, no. 3 (2024): 612–622.

34. Chang D, Truong E, Mena EA, et al., "Machine Learning Models Are Superior to Noninvasive Tests in Identifying Clinically Significant Stages of NAFLD and NAFLD-Related Cirrhosis," *Hepatology* 77, no. 2 (2023): 546–557.

35. Cholankeril G, Kramer JR, Chu J, et al., "Longitudinal Changes in Fibrosis Markers Are Associated With Risk of Cirrhosis and Hepatocellular Carcinoma in Non-Alcoholic Fatty Liver Disease," *Journal of Hepatology* 78, no. 3 (2023): 493–500.

36. Gordon SC, Kachru N, Parker E, Korrer S, Ozbay AB, Wong RJ, "Health Care Use and Costs Among Patients With Nonalcoholic Steatohepatitis With Advanced Fibrosis Using the Fibrosis-4 Score," *Hepatology Communications* 4, no. 7 (2020): 998–1011.

37. Kaplan DE, Teerlink CC, Schwantes-An TH, et al., "Clinical and Genetic Risk Factors for Progressive Fibrosis in Metabolic Dysfunction-Associated Steatotic Liver Disease," *Hepatology Communications* 8, no. 7 (2024).

38. Koch DG, Rockey DC, Litwin SS, Tedford RJ, "H2FPEF Scores Are Increased in Patients With NASH Cirrhosis and Are Associated With Post-liver Transplant Heart Failure," *Digestive Diseases and Sciences* 69, no. 8 (2024): 3061–3068.

39. Noureddin M, Mena E, Vuppalanchi R, et al., "Increased Accuracy in Identifying NAFLD With Advanced Fibrosis and Cirrhosis: Independent Validation of the Agile 3+ and 4 Scores," *Hepatology Communications* 7, no. 5 (2023).

40. Pinyopornpanish K, Al-Yaman W, Butler RS, Carey W, McCullough A, Romero-Marrero C, "Chemopreventive Effect of Statin on Hepatocellular Carcinoma in Patients With Nonalcoholic Steatohepatitis Cirrhosis," *American Journal of Gastroenterology* 116, no. 11 (2021): 2258–2269.

41. Quillin RC, 3rd, Wilson GC, Sutton JM, et al., "Increasing Prevalence of Nonalcoholic Steatohepatitis as an Indication for Liver Transplantation," *Surgery* 156, no. 4 (2014): 1049–1056.

42. Thuluvath PJ, Hanish S, Savva Y, "Waiting List Mortality and Transplant Rates for NASH Cirrhosis When Compared With Cryptogenic, Alcoholic, or AIH Cirrhosis," *Transplantation* 103, no. 1 (2019): 113–121.

43. Vieira Barbosa J, Milligan S, Frick A, et al., "Fibrosis-4 Index as an Independent Predictor of Mortality and Liver-Related Outcomes in NAFLD," *Hepatology Communications* 6, no. 4 (2022): 765–779.

44. Wang TJ, Jirapinyo P, Shah R, et al., "EUS-Guided Liver Palpation as a Screening Tool for Advanced Fibrosis and Cirrhosis in Patients With Suspected Metabolic Dysfunction-Associated Steatotic Liver Disease: A Pilot Study," *Gastrointestinal Endoscopy* 100, no. 2 (2024): 317 e311–317 e319.

45. Sanyal AJ, Shankar SS, Yates KP, et al., "Diagnostic Performance of Circulating Biomarkers for Non-Alcoholic Steatohepatitis," *Nature Medicine* 29, no. 10 (2023): 2656–2664.

46. Brandman D, Boyle M, McPherson S, et al., "Comparison of Clinical Prediction Rules for Ruling out Cirrhosis in Nonalcoholic Fatty Liver Disease (NAFLD)," *Alimentary Pharmacology & Therapeutics* 55, no. 11 (2022): 1441–1451.

47. European Association for the Study of the Liver, European Association for the Study of Diabetes, European Association for the Study of Obesity, "EASL-EASD-EASO Clinical Practice Guidelines on the Management of Metabolic Dysfunction-Associated Steatotic Liver Disease (MASLD)," *Journal of Hepatology* 81, no. 3 (2024): 492–542.

48. Harrison SA, Wong VW, Okanoue T, et al., "Selonsertib for Patients With Bridging Fibrosis or Compensated Cirrhosis Due to NASH: Results From Randomized Phase III Stellar Trials," *Journal of Hepatology* 73, no. 1 (2020): 26–39.

49. Harrison SA, Abdelmalek MF, Caldwell S, et al., "Simtuzumab is Ineffective for Patients With Bridging Fibrosis or Compensated Cirrhosis Caused by Nonalcoholic Steatohepatitis," *Gastroenterology* 155, no. 4 (2018): 1140–1153.

50. Eshraghian A, Nikeghbalian S, Dehghani M, et al., "Nonalcoholic Steatohepatitis Is the Most Rapidly Growing Indication for Liver Transplantation in Iranian Patients," *Experimental and Clinical Transplantation* 20, no. 5 (2022): 487–494.

51. Ferrarese A, Battistella S, Germani G, et al., "NASH Up, Virus Down: How the Waiting List Is Changing for Liver Transplantation: A Single Center Experience from Italy," *Medicina (Kaunas)* 58, no. 2 (2022).

52. Alkhouri N, Hanouneh IA, Zein NN, et al., "Liver Transplantation for Nonalcoholic Steatohepatitis in Young Patients," *Transplant International* 29, no. 4 (2016): 418–424.

53. Rinella ME, Satapathy SK, Brandman D, et al., "Factors Impacting Survival in Those Transplanted for NASH Cirrhosis: Data From the NailNASH Consortium," *Clinical Gastroenterology and Hepatology* 21, no. 2 (2023): 445–455 e442.

54. Doycheva I, Issa D, Watt KD, Lopez R, Rifai G, Alkhouri N, "Nonalcoholic Steatohepatitis is the Most Rapidly Increasing Indication for Liver Transplantation in Young Adults in the United States," *Journal of Clinical Gastroenterology* 52, no. 4 (2018): 339–346.
